# Effects of an App-Based Intervention on Psychological Well-Being Among Young Individuals not in Employment, Education, or Training With and Those Without Disability: Subgroup Analysis of a Randomized Controlled Trial

**DOI:** 10.2196/71367

**Published:** 2026-02-12

**Authors:** Lisa Blom, Jenny Rickardsson, Fredrik Livheim, Lene Lindberg

**Affiliations:** 1 Department of Global Public Health Karolinska Institutet Stockholm Sweden; 2 Department of Clinical Neuroscience Karolinska Institutet Stockholm Sweden

**Keywords:** not in employment, education, or training, NEET, disability, intervention, app-based, psychological health, acceptance and commitment therapy

## Abstract

**Background:**

The population of young individuals not in employment, education, or training (NEET) is highly diverse, but a common problem appears to be their mental health. NEETs due to illness or disability are of particular concern for social exclusion, but little is known of how young individuals who are NEET with and without disability make use of, and gain from, employment interventions. There is also a scarcity of research on psychological interventions and mental health outcomes among young NEETs. Acceptance and commitment therapy (ACT) has shown promising results in psychological outcomes in young adults.

**Objective:**

This study aimed to expand the knowledge on the effects of an app-based intervention built on ACT on NEETs with and without disabilities.

**Methods:**

A 2-arm randomized controlled trial was conducted in 2021, including 151 young NEETs aged 16-24 years. Participants were recruited mainly via social media platforms and through organizations working with young NEETs. The intervention group (n=77) used an app for psychological well-being with the possibility for digital group meetings for 6 weeks, and the control group (n=74) received film clips once a week. Outcomes were self-assessed through questionnaires. Statistical analyses were made using chi-square, Mann-Whitney *U* test, general linear model, and logistic regression.

**Results:**

No differences in effects on mental health were seen between the intervention and control group, neither overall nor between young NEETs with or without disability. Usage data show that 68.8% (53/77) of the participants in the intervention group downloaded the app, and 24.7% (19/77) completed all 6 modules. Effects on employment and education levels were only seen within the intervention group, where those who had completed one or more modules had a higher likelihood of being active in terms of employment and education compared to those who did not complete modules. No significant effects were seen in employment and education levels in relation to disability status. A high proportion of the participants had a disability, few were in contact with a youth employment center, and there was an overrepresentation of young women in general. Participants with disabilities had lower self-esteem, had less frequently completed high school, had less work experience, and a larger proportion had been in the NEET situation for over a year. A higher dropout were seen among participants in the intervention group and among young men.

**Conclusions:**

No effects of the app-based intervention were seen for psychological well-being between young NEETs with disabilities and those without, but the results showed potential effects on employment and education levels related to engagement in the intervention. NEETs with disabilities are of particular concern and might need additional efforts or other types of interventions than the one investigated in this study. Findings can be considered weak due to the low adherence and high attrition.

**Trial Registration:**

ISRCTN Registry ISRCTN46697028; https://www.isrctn.com/ISRCTN46697028

## Introduction

There is a global consensus on the importance of reducing the proportion of young individuals not in employment, education, or training (NEET), as demonstrated in Sustainable Development Goal 8.6 [1]. NEET status per se can be associated with long-term detriments and disadvantages such as economic scarring [2], social exclusion [3], health scarring by impacts on mental health and health behavior [4], and preterm mortality [5]. The population of NEETs is highly diverse, and its composition and characteristics vary between European countries [3]. In the case of Sweden, there is a larger proportion of short-term unemployed NEETs (35.5% vs 25.5%), a substantially higher proportion of NEETs due to illness or disability (16.1% vs 7.1%), and a lower proportion of long-term unemployed NEETs (8.5% vs 23.1%) compared to Europe as a whole [6]. NEETs due to illness or disability (defined as “young people who are not seeking employment or are not available to start a job within two weeks due to illness or disability” including individuals who cannot do paid work due to their illness or disability) are described as of particular concern for social exclusion [3].

Leaving school early or being in the NEET situation is more common among young people with disabilities compared to those without, indicating that the transition from school to work is more challenging for young individuals with disabilities [7]. A recent Organisation for Economic Co-operation and Development (OECD) report states that 8% of 15- to 29-year-olds had a disability in 2019, with a varying prevalence around the world [7]. The incidence is on the rise due to an increasing incidence of chronic depression, particularly in the Nordic countries. This trajectory was further accelerated by the COVID-19 pandemic [8] when mental health support was disrupted, schools closed or shifted to online education, daily routines and social contact were lost, and the labor market became extremely unstable [9]. Work can be a protective factor for mental health if working conditions are adequate [10].

The potential for employment interventions directed toward the NEET population has been demonstrated in a review [11], but the number of conducted trials is few and little is known about effectiveness in relation to, for example, different types of approaches or specific subgroup populations. There is also a scarcity of research on psychological interventions, including mental health outcomes. The review calls for more research on specific aspects of interventions that work and for whom, and highlights that subgroup differences to the detriment of the most disadvantaged groups were shown in some trials in relation to both effects, recruitment, and intervention engagement [11]. The need for intervention studies increasing the understanding of the effects on different subgroups of vocational and mental health support for young people who are NEET is also highlighted in a systematic review and meta-analysis on the mental health of young people who are NEET [12]. A qualitative study on experiences from a program aiming to engage young NEET showed how different elements impacted differently on different individuals [13]. Another study focusing on psychological therapy treatment outcomes of young NEETs showed that young NEETs had worse outcomes in recovery, deterioration, and attrition compared to non-NEET individuals and that the difference between the groups grew in the first few months of the COVID-19 pandemic [14]. There were also differences in outcomes within the NEET group depending on ethnicity and level of deprivation of residential areas [14]. The same study also reports an association between attending more sessions and improved outcomes [14].

Web- and app-based interventions [15] have the potential to reach populations that are normally hard to reach. The evidence around eHealth and smartphone-based psychological interventions is yet to be concluded, but has shown small positive effects on general mental health [15], anxiety and depression [15-17], and acceptance and mindfulness skills [18], and are described as promising tools to make psychotherapy more widely available even though the effects seem to be smaller than face-to-face psychotherapy. For adolescents, the evidence of these kinds of interventions is even scarcer [15]. Web-based acceptance and commitment therapy (ACT) has shown promising results in reducing stress and increasing academic buoyancy among adolescents [19] and improving psychological, emotional, and social well-being, life satisfaction, and self-esteem, and reducing symptoms of stress and depression among university students [20]. ACT aims to increase psychological flexibility, helping individuals regulate difficult thoughts and emotions while engaging in valued actions [16]. These mechanisms may be particularly relevant for NEET youth facing uncertainty, low self-esteem, or avoidance patterns, and could theoretically support both well-being and reengagement in work or education. A meta-analysis on the efficacy of smartphone-based mental health interventions called for more research investigating which aspects of interventions are effective and for whom they are beneficial [21]. Several reviews also underline the difference in effects dependent on the choice of control group, with larger effects when using a waitlist or no treatment control compared to an active control group [15,16,18]. Adherence is another aspect to take into account, where a systematic review and meta-analysis of self-guided online acceptance and commitment therapy interventions reported that 57.6% of all participants completed all modules, with adherence data ranging from 27.5% to 94.7% in the included studies [16]. Another review focusing on web-delivered acceptance and commitment therapy for mental health and well-being reported a mean adherence of 83% in terms of completing post-assessment, ranging from 48% to 100% [17]. Using reminders has been suggested to improve adherence and subsequently the effects of the interventions [15,18].

Recruitment and attrition are indeed other problems related to intervention studies on the population group NEET. There are numerous reports of the difficulty in reaching NEETs, in particular long-term NEETs and those that are not registered at the public employment services [22]. In Sweden, where this particular study took place, young women in the NEET situation have been particularly difficult to reach with interventions [23]. Social media have shown promise as a possible way to reach hard-to-reach populations [24,25] but have mostly been used for observational or cross-sectional studies and less for clinical or interventional studies [24,25]. In terms of attrition, a recent evidence and gap map of youth employment interventions rated 73.4% of the studies to be of low quality, which was most commonly related to high attrition rates or nonreporting of attrition. Another reason for the low-quality impact evaluations was large differences between intervention and control groups in baseline characteristics [26]. Furthermore, a study on an apprenticeship program directed to young NEETs showed that younger individuals and those with worse relationships with parents were more likely to drop out, underlining the need for different support approaches considering the background of the individuals [27].

In line with the research gaps identified relating to differences in how subgroups of NEETs make use of, and gain effects from, mental health interventions, the study aimed to expand the knowledge on the effects of an app-based intervention built on acceptance and commitment therapy on NEETs with and without disabilities. The following research question was considered: How do the effects (well-being, psychological distress, and employment and education level) of an app-based intervention on psychological well-being on young NEETs in Sweden vary depending on the presence of disability and moderated by background characteristics?

## Methods

### Study Design

A 2-armed parallel randomized controlled trial (RCT) was conducted between March and October 2021 to assess the feasibility and effectiveness of an app-based intervention built on ACT in comparison to film clips from YouTube dealing with mental health problems. The CONSORT-EHEALTH (Consolidated Standards of Reporting Trials of Electronic and Mobile Health Applications and Online Telehealth) guidelines for reporting RCT [28] were used (Multimedia Appendix 1).

### Inclusion and Exclusion Criteria

To be eligible for participation in the study, participants had to be 16-24 years of age, unemployed or not working or studying more than 19 hours per week. The reasons for including individuals who were active up to 19 hours per week were to include semiactive students struggling to get through high school and individuals with insecure employment. They also had to be willing and have the possibility to participate in the full intervention and have a sufficient level of Swedish to take part in the intervention. Participants who received high values on screening for depression, that is, 15 points or more on the Patient Health Questionnaire-9 (PHQ-9), were excluded and provided contact details on where to seek care. The reason for exclusion was that depression was considered to need more treatment than this study intervention could provide. The excluded participants were also offered the chance to be contacted by any of the psychologists involved in the study if they wished to have further support.

### Recruitment

The recruitment of participants from all over Sweden was done in two different phases, with the first phase of the recruitment taking place in March and April 2021, with advertisements on Facebook and Instagram. At this stage, municipalities and organizations working with NEETs were contacted and asked for support to reach out by posting on their social media, informing potential participants about the study, or having the study coordinator inform at digital group meetings. The study was also advertised on the Karolinska Institutet website, “Research subjects wanted.” When a participant declared interest in participating in the study at this stage, she or he received an email with a link to a participant information and consent form, followed by a baseline questionnaire. Despite the efforts made to reach out broadly to the target group, recruitment went slowly, which is why a new take on the recruitment strategy had to be made. Phase 2 of the recruitment started in May 2021, when the developer of the app that was tested in the study helped to create a short recruitment video that could be posted on other social media sites such as Snapchat, TikTok, and YouTube. Snapchat became the primary recruitment source. Those who declared interest in this phase were sent a text message where they were asked if the study coordinator could contact them by phone to give them more information about the study or if they preferred to be emailed. All advertisements and information regarding the study contained the logo of Karolinska Institutet.

### Randomization

Randomization was performed continuously when 20 new participants had been recruited. This number was twice the number of participants that were required for a full potential group for the digital group meetings in the intervention group (described below). Randomization was performed 1:1 by the study coordinator using the statistical program IBM SPSS by inserting the study ID of the participants and using the command “Random sample of cases,” requesting that exactly 10 out of 20 be selected. The randomization was performed 3 times, and the third randomization was used for all randomization rounds. Due to logistical reasons related to the timing of the group meetings, randomization was sometimes performed before reaching 20 new individuals. In those cases when an uneven number of individuals had been recruited, one additional person was requested to be assigned to the intervention group.

### Participants

A total of 590 young individuals declared interest in participating in the study. Of those, 193 individuals consented to participate in the study and filled in the baseline questionnaire. Due to high scores on the screening for depression, 42 individuals were excluded from the study and provided contact details for where to seek care. A total of 151 participants were randomized into either the intervention group (n=77) or the control group (n=74) and started the study.

### The Intervention

The active intervention was a youth program for increased self-compassion, self-care, mindfulness, and value-based behaviors, delivered in the 29k app (29k Foundation) [29]. The 29k app was developed by the nonprofit foundation 29k to make evidence-based tools for mental health available to as many as possible in a user-friendly manner. The youth version used in the study was in Swedish, had been piloted on young people with different backgrounds and experiences, and consisted of 6 modules that were to be used during 6 weeks. The modules included interactive material and exercises on stress, self-compassion, gratitude, relationship skills, recovery, and mindfulness exercises. In the app, additional modules were freely available for the participants to use, but no updates to the app were made, so the content remained the same throughout the trial. Participants randomized to the intervention group received individual, unique links to download the app. Participants were provided with contact details for the research team in case they needed support concerning the use of the app. Participants in the intervention group were invited to participate in weekly digital group meetings led by a trained facilitator. The facilitators did not need to be mental health professionals and got 1.5 hours of training in how to facilitate the groups. The meetings were 45 minutes long. Participation in the digital group meetings was voluntary, and the participants could choose to participate in one or several meetings or decide to use the app program on at least a weekly basis by themselves without participating in the meetings. Reminders were emailed for group meetings, and those reminders were also planned to work as reminders for using the app.

### The Control Condition

The control intervention consisted of YouTube film clips where young adults were discussing stress, mental health problems, self-esteem, and the meaning of life (Multimedia Appendix 2). An active control was chosen to reduce expectancy effects and ensure that both groups receive a structured weekly engagement. This design provides a conservative test of intervention-specific effects. Links to the film clips were sent out via email once a week for 6 weeks, and the clips were between 4 and 8 minutes long. The participants were asked to watch the video that was provided in the link and reflect for themselves. A reminder was sent out three days after the link was sent.

### Data Collection and Measures

#### Overview

Data were collected using self-assessed electronically distributed questionnaires at three timepoints: baseline (t1), postintervention (t2), and 6 months after randomization (t3). The secure web platform REDCap (Research Electronic Data Capture; Vanderbilt University) was used for overall project management and for data collection by sending out links to the questionnaires as well as reminders when the questionnaires were not completed. The questionnaire included background questions on age, sex, country of birth, physical or psychological disability or diagnosis, years of schooling, work experience, relationship with parents, trust in different societal institutions, trust in general, and how they found out about the study. The follow-up questionnaires (t2 and t3) also contained 3 free-text questions on whether the study had affected motivation, supported knowing what to do in life, and affected how the participant cares for, or commits to, others or the environment. Furthermore, the following outcome measures were included in the questionnaire.

#### Self-Esteem

Self-esteem was measured with the Swedish version of the Rosenberg self-esteem scale [30], consisting of 10 items to be answered with a 4-grade scale stretching from “strongly agree” to “strongly disagree.”

#### Well-Being

The Swedish version of the World Health Organization-Five Well-Being Index (WHO-5) [31] was used to assess well-being among the participants. The WHO-5 has five items to be answered on a 6-grade Likert scale ranging from “all the time” to “at no time.”

#### Stress

Stress was measured with the Swedish version of the Perceived Stress Scale (PSS-10) [32,33], consisting of 10 items to be answered with a 5-grade Likert scale representing the range from “never” to “very often.”

#### Anxiety

The Swedish version of the Generalized Anxiety Disorder 7-item scale (GAD-7) [34] was used to assess anxiety among the participants. The instrument consists of seven items to be answered with a 4-grade Likert scale ranging from “not at all” to “nearly every day.”

#### Depression

Depression was measured using PHQ-9 [35]. The PHQ-9 is used in Swedish health care as a screening instrument for depression and consists of 9 items to be answered with a 4-grade Likert scale representing the range from “not at all” to “nearly every day.”

#### Career Adaptabilities

Career adaptabilities are individual resources for coping with occupational challenges and tasks. The outcome was measured with a translated version of the Career Adapt-Abilities Scale - Short Form [36]. The scale consists of 12 questions that are to be answered using a 5-point Likert scale ranging from 1=“not a strength” to 5=“greatest strength.”

#### Employment or Education Status

The outcome was measured by questions on whether the participant was working, in education, or in training (3 different questions). The total time that the participants responded that they were either in employment, education, or training was compiled. A total of 20 hours or more per week was considered “active,” less than 20 hours a week was regarded as “not active.”

For the intervention group, data on app downloads, registration, and completed lessons were collected. Participation in the digital group meetings was noted by the facilitators. Given the design of the study, neither the participants nor the group leaders could be blinded to the intervention conditions. Participants received information about the 2 conditions (intervention and control) and to which group they were assigned. Group leaders provided parts of the intervention and feedback on why they were aware of the condition. Researchers had access to the assessments made online.

### Data Treatment and Analyses

Sample size was compiled based on a power of .80 and α=.05 for a moderate effect in an analysis of variance with two groups, resulting in a sample of 64 participants in each group [37]. The aim of recruitment was 180 participants to allow for a dropout of about 25%.

Differences in background data of the participants in the intervention and control groups were tested with the chi-square for categorical variables and the Mann-Whitney *U* test for continuous variables due to nonnormality of the variables. The Pearson correlation test was used to assess the correlation between the total score of the Career Adapt-Abilities Scale and completion of modules in the app for the participants in the intervention group.

Composite scores were compiled for the outcomes “well-being” and “psychological distress” using *z*-scores. For well-being, the *z* scores from the instruments WHO-5 and the Rosenberg self-esteem scale were summed into one composite variable. For psychological distress, the *z* scores from the instruments GAD-7, PSS-10, and PHQ-9 were summed. Composite scores were chosen to reduce the number of statistical tests.

A general linear model (GLM) was used to analyze the differences between t1 and t2 for the composite scores of well-being and psychological distress. Analyses were made using both per protocol (PP) and intention to treat (ITT) approach, where missing values in the ITT analysis were replaced with series means of the intervention and control group. The ITT approach assumed data were missing at random at the level of the treatment group. Differences in well-being and psychological distress (t2-t1), respectively, were used as dependent variables in each model. Group, gender, and disability were used as fixed factors in the GLM model. The interaction effect between those variables was included in the tables. Country of birth was not included as a fixed factor since the number of participants born abroad was small. Binary logistic regression was used for the analyses of employment and education status at t3. IBM SPSS (version 28) was used for all analyses.

### Ethical Considerations

The study was approved by the Swedish Ethical Review Authority (Dnr 2020-03952). All participants were informed about the study and their rights and signed a digital informed consent form before participation. Data collected are stored in secure databases at Karolinska Institutet and presented at the group level to minimize the risk of identifying specific participants. Participants were reimbursed with a gift card of 750 SEK after completing the final questionnaire to cover the time used for the study.

## Results

**Figure 1:**
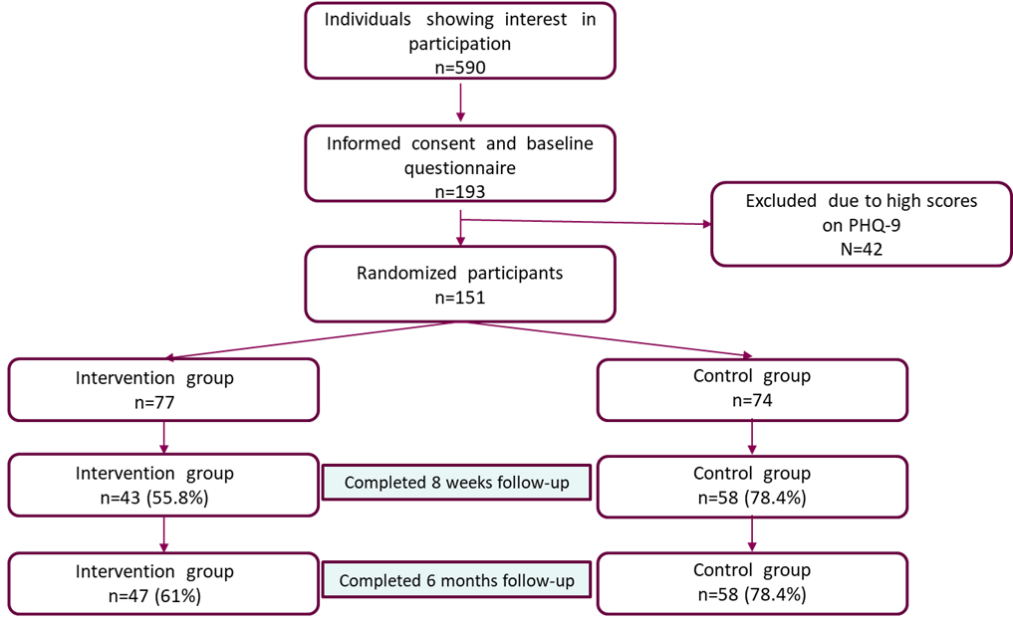
Flowchart of the recruitment and the participants. PHQ-9: Patient Health Questionnaire-9.

Of the 151 participants who participated in the study (Figure 1), almost three-quarters were young women (Table 1). The mean age of the participants was 20 (SD 2) years, and 9 out of 10 were born in Sweden. Almost half of the participants responded that they had a physical or psychiatric disability or diagnosis (hereafter mentioned as “disability”). More than half of those had several different disabilities, and the most common ones were psychiatric disabilities such as attention deficit hyperactivity disorder (ADHD; n=35), depression (n=25), generalized anxiety (n=20), and autism (n=15). The majority of the participants had passed ninth grade, and about 6 out of 10 had completed high school. Participants came from all parts of Sweden, about half from the middle part, a third from southern Sweden, and the rest from the northern part of Sweden. The largest proportion of participants came from large cities or municipalities nearby, followed by medium-sized towns and nearby municipalities, while 18.5% (28/151) of participants came from small towns or rural municipalities. Slightly more than half of the participants had been outside of employment, education, or training for 3-12 months. Only 21.9% (33/151) were in contact with a youth employment center or other activity directed to NEET youths. No significant differences in terms of background characteristics were found between the intervention and the control group.

**Table 1:** Background characteristics of the participants at baseline assessment for the intervention and control groups, and for participants with disabilities and no disability.

		All (N=151), n (%)	Intervention (N=77), n (%)	Control (N=74), n (%)	*P* value^a^		Disability (N=75), n (%)		No disability (N=76), n (%)		*P* value^a^	
**Gender, n (%)**						.26^b^						.28^b^
	Women	113 (74.8)	56 (72.7)	57 (77)			60 (80)		53 (69.7)			
	Men	33 (21.9)	20 (26)	13 (17.6)			14 (18.7)		19 (25)			
	Other or don’t know	5 (4.8)	—^c^	—^c^			—^c^		—^c^			
**Group**						—^d^						.37
	Intervention	77 (51)	—^d^	—^d^			41 (54.7)		36 (47.4)			
	Control	74 (49)	—^d^	—^d^			34 (45.3)		40 (52.6)			
Age (years), mean (SD)		20.0 (2.0)	20.0 (2.0)	20.0 (2.1)	.98		19.9 (2.3)		20.1 (1.8)		.66	
**Birth country**						.54						.62
	Sweden	135 (89.4)	70 (90.9)	65 (87.8)			68 (90.7)		67 (88.2)			
	Other than Sweden	16 (10.6)	7 (9.1)	9 (12.2)			7 (9.3)		9 (11.8)			
**Disability**						.37						—^d^
	Yes	75 (49.7)	41 (53.2)	34 (45.9)			—^d^		—^d^			
	No	76 (50.3)	36 (46.8)	40 (54.1)			—^d^		—^d^			
Years of education, mean (SD)		11.7 (2.0)	11.9 (1.6)	11.5 (2.4)	.59		11.3 (2.3)		12.1 (1.6)		.08	
**Graduated ninth grade**						.06						.53
	Yes	139 (92.1)	74 (96.1)	65 (87.8)			68 (90.7)		71 (93.4)			
	No or don’t know	12 (7.9)	—^c^	9 (12.2)			7 (9.3)		5 (6.6)			
**Completed high school**						.77						.01
	Yes	88 (58.3)	44 (57.1)	44 (59.5)			36 (48)		52 (68.4)			
	No	63 (41.7)	33 (42.9)	30 (40.5)			39 (52)		24 (31.6)			
**Part of the country**						.46						.34
	Southern Sweden	51 (33.8)	29 (37.7)	22 (29.7)			24 (32)		27 (35.5)			
	Mid Sweden	78 (51.7)	36 (46.8)	42 (56.8)			37 (49.3)		41 (53.9)			
	Northern Sweden	20 (13.2)	11 (14.3)	9 (12.2)			13 (17.3)		7 (9.2)			
	Information missing	—^c^	—^c^	—^c^			—^c^		—^c^			
**Type of municipality**						.95						.15
	In or near large cities	66 (43.7)	33 (42.9)	33 (44.6)			28 (37.3)		38 (50)			
	In or near medium-sized towns	55 (36.4)	29 (37.7)	26 (35.1)			28 (37.3)		27 (35.5)			
	Small towns or rural	28 (18.5)	14 (18.2)	14 (18.9)			18 (24)		10 (13.2)			
	Information missing	—^c^	—^c^	—^c^			—^c^		—^c^			
**Time in NEET** ^e^ **status**						.88						.01
	0-2 months	34 (22.5)	17 (22.1)	17 (23)			17 (22.7)		17 (22.4)			
	3-12 months	82 (54.3)	41 (53.2)	41 (55.4)			33 (44)		49 (64.5)			
	13 months or more	29 (19.2)	16 (20.8)	13 (17.6)			21 (28)		8 (10.5)			
	Information missing	6 (4)	—^c^	—^c^			—^c^		—^c^			
**Contact with the youth employment center or other activity working with NEETs**						.64						.88
	Yes	33 (21.9)	18 (23.4)	15 (20.3)			16 (21.3)		17 (22.4)			
	No	118 (78.1)	59 (76.6)	59 (79.7)			59 (78.7)		59 (77.6)			
**Previous work experience**						.23						.005
	Yes	97 (64.2)	53 (68.8)	44 (59.5)			40 (53.3)		57 (75)			
	No	54 (35.8)	24 (31.2)	30 (40.5)			35 (46.7)		19 (25)			

^a^*P* values were calculated by chi-square or Mann-Whitney *U* tests.

^b^Analysis only including women or men categories.

^c^Not displayed as n<5.

^d^Not applicable.

^e^NEET: not in employment, education, or training.

Some statistically significant differences were found in background characteristics between those who reported a disability and those who did not (Table 1). A lower proportion of those with a disability had completed high school and had previous work experience compared to those without disability. Participants with disabilities also had a higher proportion of 13 months or more with NEET status.

There were no significant differences between the intervention and the control group in self-reported health at baseline (Multimedia Appendix 3). The participants had a mean value of 45.7 in the instrument WHO-5, which can be regarded as relatively low considering that values below 52 points indicate further investigation when used in health care [31]. The mean value of the participants on self-esteem, 14.5 points, was on the limits of low and normal self-esteem. About half (74/151, 49.7%) of the participants had values indicating mild anxiety, while 27% (41/151) had values indicating moderate or severe anxiety. The stress levels of the participants were classified as moderate in 74.8% (113/151), and 11.9% (18/151) had values indicating high stress levels. About 57% (86/151) had moderate depressive symptoms, and the remaining participants had either no, minimal, or mild depressive symptoms (those with more severe symptoms had been excluded).

The participants who reported a disability had a significantly lower mean score on self-esteem compared to those without disability (12.0 vs 16.1). No other significant differences were observed (Multimedia Appendix 3).

Table 2 shows that about 30% (46/151) of the participants did not complete the final questionnaire at 6 months and were considered to have dropped out. There was a significantly higher dropout in the intervention group compared to the control group, and among young men compared to young women. There was also a higher proportion of those born outside of Sweden who dropped out, but the difference was not significant. No difference in dropout was found in the other background factors. Looking further into the intervention group, a significantly higher share of the young men dropped out compared to the young women in the intervention group (12/77, 15.6% vs 18/77, 23.4%; *χ*^2^_1_=.03). No other significant differences were found in terms of dropout in the intervention group related to age, country of birth, or disability status.

**Table 2:** Proportion of participants who did and did not complete the final follow-up questionnaire 6 months post randomization.

			Participants, n		Proportion that completed, n (%)		Proportion that did not complete, n (%)		Chi-square (*df*)	
All			151		105 (69.5)		46 (30.5)			
**Group**										
	Intervention	77		47 (61)		30 (39)		.02 (1)		
	Control	74		58 (78.4)		16 (21.6)				
**Gender**										.01^a^ (1)
	Women	113		84 (74.3)		29 (25.7)				
	Men	33		16 (48.5)		17 (51.5)				
	Other or don’t know	5		5 (100)		0 (0)				
**Age**										.79 (1)
	16-19	60		41 (68.3)		19 (31.7)				
	20-24	91		64 (70.3)		27 (29.7)				
**Country of birth**										.09 (1)
	Sweden	135		97 (71.9)		38 (28.1)				
	Other country	16		8 (50)		8 (50)				
**Disability**										.68 (1)
	Yes	75		51 (68)		24 (32)				
	No	76		54 (71.1)		22 (28.9)				
**Relationship with mother**										.70 (1)
	Very good or good	105		72 (68.6)		33 (31.4)				
	Ok to very bad or no answer	46		33 (71.7)		13 (28.3)				
**Relationship with father**										.45 (1)
	Very good or good	62		41 (66.1)		21 (33.9)				
	Ok to very bad or no answer	89		64 (71.9)		25 (28.1)				
**Time outside of studies or employment**										.67 (2)
	<3 months	34		24 (70.6)		10 (29.4)				
	3-12 months	82		55 (67.1)		27 (32.9)				
	>12 months	29		22 (75.9)		7 (24.1)				
**Recruitment phase**										.41 (1)
	First phase	33		21 (63.6)		12 (36.4)				
	Second phase	118		84 (71.2)		34 (28.8)				

^a^Chi-square analysis only including women and men categories.

Usage data from the app show that about 7 out of 10 participants in the intervention group downloaded and registered in the app (Table 3). Of those that registered, about 87% (46/53) consented to having their usage followed. Slightly more than half of the participants did not complete any modules in the app. About 27% (21/77) of the participants completed 4 or more modules, and the same percentage participated in one or several digital group meetings. There were no significant differences in usage between those with disabilities and those without disabilities. Multimedia Appendix 4 displays some background and health-related characteristics of the participants who completed any modules in the app and those who did not complete any. Among those who did not complete any modules, there was a higher proportion of participants born outside of Sweden, a lower proportion of participants reporting that they trust other people in general, and a higher proportion who were in contact with a youth employment center or other activity toward young NEETs. No significant differences were seen for the other characteristics. A sensitivity analysis examining the correlation between the score on the Career Adapt-Abilities Scale from the participants in the intervention group at the beginning of the study and whether they completed modules or not resulted in a significant negative correlation (Pearson correlation coefficient *r*=–.31; *P*=.01). Hence, participants who completed modules tended to score lower on the scale than those that had not completed modules. In terms of time dedicated per week for the study, 28.4% (21/74) of the participants in the control group did not answer the question, 33.8% (25/74) answered that they had used 1-15 minutes per week, 24.3% (18/74) had used 16-30 minutes per week and 13.5% (10/74) had used more than 30 minutes per week. In the intervention group, 49.4% (38/77) did not answer, 9.1% (7/77) of the participants answered 1-15 minutes, 11.7% (9/77) had used 16-30 minutes per week, and 29.9% (23/77) had used more than 30 minutes per week for the study.

**Table 3:** Usage of the app in the intervention group, total, and depending on disability status.

			Intervention (n=77), n (%)		Disability (n=41), n (%)		No disability (n=36), n (%)		Chi-square (*df*)	
**Registered**										.17 (1)
	Yes	53 (68.8)		31 (75.6)		22 (61.1)				
	No	24 (31.2)		10 (24.4)		14 (38.9)				
**Completed modules**										.73 (1)
	0	43 (55.8)		23 (56.1)		20 (55.6)				
	1-3	13 (16.9)		8 (19.5)		5 (13.9)				
	4 or more	21 (27.3)		10 (24.4)		11 (30.6)				
**Completed all 6 modules**										.26 (1)
	Yes	19 (24.7)		8 (19.5)		11 (30.6)				
	No	58 (75.3)		33 (80.5)		25 (69.4)				
**Participation in group meetings**										.68 (1)
	Yes	21 (27.3)		12 (29.3)		9 (25)				
	No	56 (72.7)		29 (70.7)		27 (75)				

Comments in the free-text answers were similar for the participants with and without disabilities in the intervention group. Some comments were raised about using the app, which made them feel less alone, as it was comforting to hear others having similar thoughts and feelings. Other comments were that they appreciated the discussions with the group leaders during the group discussions and the exercises to work on in between. Some participants also described how they valued the exercises provided in the app, such as meditation, and that some exercises had triggered self-reflection and awareness about how they feel. Other participants expressed that they did not perceive that the app had changed their motivation. A few participants wished for a longer duration of the intervention.

The GLM analyses made PP showed no significant differences for improvement of psychological distress or well-being between the intervention and the control group, irrespective of disability status or gender (Table 4). The ITT analysis on change in well-being indicated a tendency (*P*=.08) where the intervention group had a more positive change in well-being between baseline and directly after the intervention compared to the control group. No interaction effects were observed.

When focusing on the intervention group and their exposure to the intervention using both PP and ITT analysis, there were no significant differences between those who completed one or several modules in the app compared to those who did not complete any (Table 5). Similar results were observed when looking at those who participated in the group meetings (Table 6).

The analyses regarding employment and education level of the participants 6 months after randomization did not yield any significant results when including the full sample, nor when comparing the control group to those in the intervention group that had completed any modules in the app (Table 7).

The analyses regarding employment and education level of the participants 6 months after randomization did not yield any significant results when including the full sample, nor when comparing the control group to those in the intervention group that had completed any modules in the app (Table 7).

**Table 4:** Change in psychological distress and well-being between baseline and postintervention (per protocol and intention to treat) for the intervention and control group for all participants, depending on disability status and gender.

Variables			Groups				GLM^a^ results													
			Intervention, mean (SD)		Control, mean (SD)		Main						Interaction							Partial eta squared
							*F* test (*df*)			*P* value			*F* test (*df*)			*P* value				
**Psychological distress (PP^b^)**								0.356 (1, 92)			.55			0.097 (1, 83)			.76			0.001
	All (n=94)	–0.14 (0.93)		–0.02 (0.92)																
**Disability (n=94)**								0.771 (1, 90)			.38			—^c^			—			—
	Yes	–0.01 (1.05)		0.03 (0.85)																
	No	–0.26 (0.79)		–0.06 (0.98)																
**Gender (n=91)**								0.181 (1, 87)			.67			—			—			—
	Women	–0.05 (0.92)		–0.03 (0.93)																
	Men	–0.30 (0.84)		0.00 (0.79)																
**Psychological distress (ITT^d^)**								2.081 (1, 149)			.15			0.083 (1, 138)			.77			0.001
	All (n=151)	–0.12 (0.98)		0.12 (1.02)																
**Disability (n=151)**								0.751 (1, 147)			.39			—			—			—
	Yes	–0.03 (1.04)		0.17 (0.98)																
	No	–0.22 (0.92)		0.08 (1.06)																
**Gender (n=146)**								0.081 (1, 142)			.78			—			—			—
	Women	–0.09 (1.02)		0.11 (1.04)																
	Men	–0.08 (0.82)		0.21 (0.99)																
**Well-being (PP)**								2.183 (1, 95)			.14			1.415 (1, 86)			.24			0.016
	All (n=97)	0.20 (0.86)		–0.08 (0.98)																
**Disability (n=97)**								1.329 (1, 93)			.25			—			—			—
	Yes	0.22 (0.98)		–0.36 (1.04)																
	No	0.18 (0.73)		0.12 (0.89)																
**Gender (n=94)**								0.000 (1, 90)			.99			—			—			—
	Women	0.18 (0.86)		–0.07 (0.97)																
	Men	0.26 (0.99)		–0.16 (1.12)																
**Well-being (ITT)**								3.038 (1, 149)			.08			0.040 (1, 138)			.84			0.000
	All (n=151)	0.14 (0.90)		–0.14 (1.06)																
**Disability (n=151)**								1.103 (1, 147)			.29			—			—			—
	Yes	0.17 (0.94)		–0.36 (1.13)																
	No	0.10 (0.86)		0.04 (0.98)																
**Gender (n=146)**								0.005 (1, 142)			.94			—			—			—
	Women	0.13 (0.92)		–0.15 (1.08)																
	Men	0.15 (0.89)		–0.20 (1.17)																

^a^GLM: general linear model.

^b^PP: per protocol.

^c^Not available.

^d^ITT: intention to treat.

**Table 5:** Change in psychological distress and well-being between baseline and postintervention (per protocol and intention to treat) for those in the intervention group who completed one or more modules and those who did not complete any modules for the whole intervention group, depending on disability status and gender.

Variables			Intervention group					GLM^a^ results									
			Completed one or more modules, mean (SD)		Did not complete any module, mean (SD)		Main					Interaction					Partial eta squared
							*F* test (*df*)			*P* value		*F* test (*df*)		*P* value			
**Psychological distress (PP^b^)**							1.303 (1, 39)			.26		—^c^		—		—	
	All (n=41)	–0.26 (0.71)		0.08 (1.22)													
**Disability (n=41)**							0.998 (1, 37)			.32		—		—		—	
	Yes	–0.28 (0.73)		0.34 (1.33)													
	No	–0.25 (0.71)		–0.30 (1.03)													
**Gender (n=40)**							0.174 (1, 36)			.68		—		—		—	
	Women	–0.21 (0.65)		0.23 (1.24)													
	Men	–0.49 (0.94)		0.17 (0.31)													
**Psychological distress (ITT^d^)**							3.130 (1, 75)			.08		0.225 (1, 68)		.64		0.003	
	All (n=77)	–0.33 (0.76)		0.06 (1.10)													
**Disability (n=77)**							0.597 (1, 73)			.44		—		—		—	
	Yes	–0.30 (0.80)		0.19 (1.16)													
	No	–0.37 (0.75)		–0.09 (1.04)													
**Gender (n=76)**							0.009 (1, 72)			.92		—		—		—	
	Women	–0.35 (0.67)		0.14 (1.22)													
	Men	–0.29 (1.11)		0.03 (0.65)													
**Well-being (PP)**							0.774 (1, 40)			.38		—^c^		—		—	
	All (n=42)	0.29 (0.84)		0.04 (0.90)		—											
**Disability (n=42)**							0.005 (1, 38)			.94		—		—		—	
	Yes	0.43 (0.88)		–0.09 (1.09)													
	No	0.15 (0.81)		0.23 (0.56)													
**Gender (n=41)**							0.037 (1, 37)			.85		—		—		—	
	Women	0.24 (0.80)		0.07 (0.99)													
	Men	0.50 (1.08)		–0.36 (0.39)													
**Well-being (ITT)**							2.379 (1, 75)			.13		1.400 (1, 68)		.24		0.020	
	All (n=77)	0.31 (0.89)		–0.00 (0.90)													
**Disability (n=77)**							0.162 (1, 73)			.69		—		—		—	
	Yes	0.40 (0.93)		–0.01 (0.93)													
	No	0.21 (0.85)		0.01 (0.88)													
**Gender (n=76)**							0.118 (1, 72)			.73		—		—		—	
	Women	0.28 (0.81)		–0.02 (1.00)													
	Men	0.43 (1.20)		0.01 (0.67)													

^a^GLM: general linear model.

^b^PP: per protocol.

^c^Interaction could not be computed due to too few participants in some categories.

^d^ITT: intention to treat.

**Table 6:** Change in psychological distress and well-being between baseline and postintervention (per protocol and intention to treat) for those in the intervention group who attended or did not attend group meetings for the whole intervention group, and depending on disability status and gender.

Variables			Intervention group					GLM^a^ results										
			Attended group meetings, mean (SD)		Did not attend group meetings, mean (SD)		Main						Interaction					Partial eta squared
							*F* test (*df*)			*P* value		*F* test (*df*)			*P* value			
**Psychological distress (PP^b^)**							0.054 (1, 39)			.82		0.785 (1, 32)			.38		0.024	
	All (n=41)	–0.09 (1.20)		–0.16 (0.73)														
**Disability (n=41)**							1.396 (1, 37)			.25		—^c^			—		—	
	Yes	0.31 (1.44)		–0.21 (0.72)														
	No	–0.50 (0.80)		–0.11 (0.78)														
**Gender (n=40)**							.64 (1, 36)			.43		—			—		—	
	Women	0.03 (1.23)		–0.10 (0.67)														
	Men	–0.64 (1.05)		–0.05 (0.70)														
**Psychological distress (ITT^d^)**							0.001 (1, 75)			.98		0.068 (1, 68)			.79		0.001	
	All (n=77)	–0.11 (1.28)		–0.12 (0.86)														
**Disability (n=77)**							3.819 (1, 73)			.06		—			—		—	
	Yes	0.37 (1.36)		–0.19 (0.84)														
	No	–0.75 (0.87)		–0.04 (0.88)														
**Gender (n=76)**							0.247 (1, 72)			.62		—			—		—	
	Women	–0.02 (1.32)		–0.13 (0.87)														
	Men	–0.47 (1.21)		0.02 (0.72)														
**Well-being (PP)**							0.225 (1, 40)			.64		0.595 (1, 33)			.45		0.018	
	All (n=42)	0.28 (0.97)		0.15 (0.80)														
**Disability (n=42)**							0.0125 (1, 38)			.88		—			—		—	
	Yes	0.06 (1.11)		0.31 (0.93)														
	No	0.50 (0.83)		–0.04 (0.60)														
**Gender (n=41)**							0.131 (1, 37)			.72		—			—		—	
	Women	0.18 (0.87)		0.17 (0.87)														
	Men	0.69 (1.48)		–0.07 (0.42)														
**Well-being (ITT)**							0.186 (1, 75)			.67		0.903 (1, 68)			.34		0.013	
	All (n=77)	0.21 (1.01)		0.11 (0.86)														
**Disability (n=77)**							0.797 (1, 73)			.38		—			—		—	
	Yes	–0.12 (1.01)		0.29 (0.90)														
	No	0.66 (0.87)		–0.09 (0.79)														
**Gender (n=76)**							0.297 (1, 72)			.59		—			—		—	
	Women	0.14 (0.87)		0.12 (0.95)														
	Men	0.51 (1.59)		0.06 (0.66)														

^a^GLM: general linear model.

^b^PP: per protocol.

^c^Not available.

^d^ITT: intention to treat.

**Table 7:** Logistic regression on being active 20 hours or more per week, 6 months post randomization, comparing control versus intervention group, control group vs those in the intervention group who completed one or more modules, and within the intervention group, depending on completion of modules.

				B (SE)		Odds ratio (95% CI)		*P* value
**Control vs intervention group (n=107)**								
	**Crude**							
		Intervention	0.11 (0.40)		1.12 (0.51-2.42)		.78	
	**Adjusted**							
		Intervention	0.15 (0.42)		1.17 (0.52-2.63)		.71	
		Women	0.19 (0.56)		1.21 (0.41-3.62)		.73	
		Disability	–0.70 (0.42)		0.50 (0.22-1.12)		.09	
**Control vs those in the intervention group who completed one or more modules (n=86)**								
	**Crude**							
		Intervention	0.75 (0.51)		2.12 (0.78-5.77)		.14	
	**Adjusted**							
		Intervention	0.81 (0.53)		2.25 (0.79-6.37)		.13	
		Women	0.14 (0.64)		1.15 (0.33-4.01)		.82	
		Disability	–0.83 (0.48)		0.43 (0.17-1.11)		.08	
**Only including the intervention group (n=49)**								
	**Crude**							
		Completed	1.39 (0.62)		4.00 (1.19-13.50)		.03	
	**Adjusted**							
		Completed	1.59 (0.68)		4.89 (1.30-18.38)		.02	
		Women	0.93 (0.87)		2.54 (0.46-14.02)		.29	
		Disability	–0.43 (0.67)		0.65 (0.18-2.39)		.52	

## Discussion

### Principal Findings

The study aimed to expand the knowledge on the effects in terms of well-being, psychological distress, and employment and education level of an app-based intervention on NEETs with and without disabilities. The main findings of the study are that no differences were seen overall between the intervention and control groups, irrespective of whether the participants had completed a module in the app or not. No significant differences were found regarding the effect of being active in terms of employment or education, 20 hours or more per week, 6 months post randomization, except within the intervention group, where those who had completed one or several modules in the intervention were more likely to be active compared to those who did not complete any.

The findings could be related to those described in the review of Mawn et al [11], showing varying results from the interventions, and where high-contact interventions seemed to be most effective. Participation in the digital group meetings offered to the intervention group was low in general, and it might be worth considering if other formats would suit the target group better, in particular for participants with a disability. Other studies have presented differences in effects related to, for example, gender, ethnicity, and age [11,14]. Only gender was tested in this study since the material did not allow for examining ethnicity, and the age group was too narrow, but the results on intervention effects did not differ across gender. When reflecting about the effects of the app, it is important to keep in mind that the app is health promotive in the sense that it is developed to enhance the participant’s well-being rather than to treat depression or anxiety and the target group excludes those with symptoms of severe depression, all of which are factors that could make it more difficult to detect positive effects of the intervention. Likewise, being a health-promotive app, it was not designed specifically for the purpose of getting young people into employment or education and was not intended as a stand-alone solution to replace traditional employment services but rather as a complement to other services. Indeed, several reviews emphasize the need for multicomponent strategies to cover the complex needs of the diverse population of NEETs [11,38,39]. Digital innovations could be a way to provide scalable and flexible support to young NEETs as an add-on to other activities, both focusing on psychological support and skills acquisition. One example of that is a clinically integrated online platform for young NEETs with mental health difficulties in Australia that showed positive work and study outcomes. The participants appreciated not having to travel and described the online mode as less confronting compared to in-person support, although some would have preferred meeting in-person [40]. Other studies have shown promising results from digital interventions such as the use of social media as a platform for skill acquisition [41] and a game-based positive psychology intervention [42] to support and engage young NEETs. A meta-analysis of the effectiveness of gamified interventions for mental health promotion showed positive effects on both mental wellness and psychological symptoms [43]. However, several studies on NEET emphasize the role of a mentor to handle challenges that arise [44,45], in particular for NEETs with mental health challenges or NEETs experiencing other vulnerabilities, where often more intensive strategies are needed [38]. A sustainable contact with a qualified practitioner where the relationship is built on trust and close collaboration with the young NEET and where interventions include psychological support and social skills development are identified as important features for the transition into employment for NEETs with additional vulnerabilities [38]. A Swedish study on workplace-oriented rehabilitation found, on the other hand, a model with personnel-intensive support with a clear and early focus on work to be more effective in getting young NEETs into employment compared to another personnel-intensive support model with a more holistic approach [46]. The diversity of NEETs and the complex flora of needs and interventions provides an additional argument for why digital interventions could be more suitable as a complementary intervention, enhancing other types of contacts and interventions when multicomponent interventions are recommended [38]. Some of the qualitative feedback that came from the participants in the intervention group mentioned how the use of the app made them feel less alone by knowing that others have similar thoughts and feelings. Others mentioned the importance of the discussion with the group leader during the digital group meeting. The feedback shows the potential for digital innovations to provide psychological support in a suitable manner for some participants, appreciating the digital format as a complement to the face-to-face-format although other participants described that the app did not result in any change. Whether this preference could be related to specific disabilities among the participants remains unclear, but participants with and without disabilities brought up similar issues in the free text. The heterogeneity of disabilities among the participants and the fact that the majority of those with disabilities had multiple diagnoses in our study make it difficult to draw any conclusions from the findings on the appropriateness across diverse disabilities. Further studies of a more specific nature in terms of target group and specific diagnoses could increase understanding in this area.

A previous study looking at psychological treatment outcomes found an association between improved outcomes and attending more sessions [14]. In this study, no differences in psychological outcomes were found related to completing modules in the intervention or not. However, there was a higher likelihood of being in employment or engaged in studies 6 months post randomization in this study for those in the intervention group who completed modules in the intervention compared to those who did not. This finding can indicate the importance of further completing the intervention, or at least motivating the participants to give it a try. It is also possible that the results in this study could be related to differences in participant characteristics in the sense that characteristics facilitating completing the intervention, such as, for example, motivation, could also facilitate the process of seeking employment or studies. Motivation was not measured among the participants, but career adaptability has been associated with career motivation [47]. The results from the correlation analysis between career adaptabilities and completion of modules showed a negative correlation, where those with high scores on the career adaptabilities scale tended not complete modules to a higher extent and vice versa, which was a bit surprising. These findings should be interpreted with caution, but a possible explanation could be that the group with high career adaptability had expectations on the app that were not met. Another interpretation is that the app could be a good complement to those with lower career adaptability. The data at hand and the small sample of the intervention group restrict the possibilities to examine the underlying reasons for this further, and it is an area for future research. No significant differences in being active in terms of employment or education, 20 hours or more per week, were found between the intervention and the control groups. Keeping in mind that a meta-analysis of 3 high-intensity interventions directed toward NEETs resulted in a very low increase in employment for the intervention group [11], the intervention studied in this paper is of a lower intensity and, as mentioned previously, was not designed specifically to get people into employment. Additionally, this study had an active control group, which could make it more difficult to detect intervention effects compared to having a waitlist or passive control group, as has been mentioned in previous research [15,16,18,43]. In the same vein, our choice of having the cut-off at 20 hours per week as both inclusion criteria for the study (19 hours or less per week) and as an outcome for being active (20 hours or more per week) could be debated. Similar cutoffs have been used earlier in combination with other measures [48,49], but the diverse flora of outcome measures used in studies focusing on NEETs underline the need to develop standardized outcome measures for reengagement [50]. In Sweden, like in many other countries, poor school results or high absence from school are risk factors for becoming NEET, and insecure employment is common among young individuals [51]. Having a “generous” inclusion criterion that also includes young semiactive individuals embeds for capturing individuals early on in their career in a health-promotive approach. It can also be underlined that the majority of participants in our study were at the extremes in terms of the outcome of being active, three out of four were either active 0 hours or 30 hours or more per week, 6 months post intervention.

Focusing on the participants that were recruited, the larger proportion were young women. This was a bit surprising since the gender division of NEETs in Sweden is relatively equal, with a slightly higher representation of men [6], and women have previously been reported to be difficult to recruit [23]. Young men were also overrepresented among those who dropped out. A scoping review of the use of social media for recruitment to medical research studies did not find a gender difference in the recruited participants [25], and Snapchat, which was the primary recruitment channel during the second recruitment phase when the majority of the participants were recruited, has a relatively equal use over genders [52]. However, a review of digital mental health interventions found that men were less likely to complete interventions compared to women and calls for interventions catering to the interests of young men [53]. It is possible that the intervention or the advertisements were more appealing to young women, even though the app and the short recruitment video were designed to appeal to all genders. Gamified interventions have shown promise for promoting mental health across genders, with an even greater potential effect on the reduction in anxiety symptoms in samples with a higher proportion of young men [43]. It is possible that using a gamified type of intervention could have resulted in increased recruitment and attainment of young men.

There was also a substantial proportion of those recruited who had a disability (about half), which is much higher than the general prevalence of 8% stated in an OECD report [7] and higher than the estimated 16% among NEETs in Sweden [6]. This high proportion of participants with a disability could imply that the type of intervention is particularly appealing to those with a disability, even though some adjustments have to be made for it to have an effect. As mentioned above, the heterogeneity of disabilities, as well as the high proportion of participants with multiple disabilities, complicated the interpretation of the findings in relation to disability status. The group of participants with a disability had nevertheless less frequently completed high school, which confirms the previous reports of a higher risk for school dropout among persons with, for example, depression, anxiety, or ADHD [54]. Previous reports of a difficult transition from school to work among individuals with disabilities [7] are also echoed in the findings of this study, where fewer participants with disabilities had previous working experience, and a larger proportion had been in the NEET situation for over a year. Participants with disabilities also had significantly lower self-esteem compared to those without. These findings call for further investigation of how the education system can be adjusted to meet the needs of all children and young people to lay the ground for a successful transition from school to work life. Working toward good health irrespective of disability status should be a priority in a society where all people should be entitled to be included and given possibilities for an engaged and rich life.

Overall, only 1 out of 5 participants had contact with an employment center, and it might be that this type of intervention, in combination with recruitment via social media, facilitates reaching populations that are normally difficult to reach with conventional interventions directed toward NEETs. Self-selection bias is a well-known risk when recruiting based on the fact that participants have volunteered for the study, and it might be that the recruitment strategy also attracted technology savvy participants. Furthermore, the recruiting procedure using both social media and affiliated organizations could also imply selection biases, but most probably of different kinds since the two approaches are likely to reach slightly different target groups. A large proportion of those recruited via social media had not been in contact with employment centers or similar activities, whereas those recruited via affiliated organizations had been informed about the study due to the contact they already had. The use of different approaches for recruiting was chosen to broaden the variety of young NEETs and to increase the possibility of also reaching those who had not been in contact with employment centers. Reaching participants at the beginning of their NEET journey could be a window of opportunity to prevent them from staying in the NEET situation for a long time, since research shows that individuals starting their career as unemployed are more likely to become unemployed later [55].

A major problem in the study was adherence, where slightly more than half of the participants in the intervention group did not complete any module in the app. Among those who did not complete any modules, there was also an overrepresentation of participants born outside of Sweden, a lower proportion of participants reporting that they trust other people in general, and a higher proportion being in contact with youth employment centers. In terms of time dedicated to the study per week, the intervention group had larger proportions on the “extremes” with a large proportions of participants who did not answer, but also a larger proportion of participants who had used more than 30 minutes per week for the study compared to the control group. Just by looking at what was required by the participants from the start, the time for the control arm each week was considerably shorter compared to the intervention arm, with the film clips being 4-8 minutes long. The higher time burden in the intervention arm compared with the control condition likely contributed to lower adherence and should be viewed as a structural feature of the design. Adherence is a problem seen in other similar studies based on smartphone or online interventions [16,56]. To improve adherence to mental health apps, actions such as involving end users in the development and thorough usability testing, as well as including clinician or peer support, have been suggested [56]. The apps that were tested in this study were developed with continuous feedback and input from end-users and underwent usability testing. It also included the digital group meetings that were intended to provide peer support, although these were not used much by the participants and sometimes even seemed to scare some of the participants off. The anonymity of the online context is indeed an appreciated feature that needs to be considered and balanced with the advantage of including social support or interaction with health professionals within an app [53].

High dropout is a common problem in both digital mental health interventions [56] and youth employment interventions [26]. In this study, 3 out of 10 participants did not complete the follow-up questionnaire 6 months post randomization, which is a relatively expected share given the target group, although slightly above the expected 25%. Efforts were made from the start to increase retention by using reminders as well as providing reimbursement for participation in the study, actions that have been suggested to reduce attrition [56]. We also changed the recruitment procedure along the way to have a mixed recruitment procedure involving both online registration, text message, and phone contact, but many participants actually chose to only use the online registration because they did not feel comfortable talking on the phone. Apart from a higher dropout among young men (discussed above), there was a higher dropout among participants in the intervention group. This has been seen in other studies using inactive control group but, to our knowledge, not in studies using active control groups [56], such as this particular study. The high drop-out in the intervention group is likely related to the experienced higher demand and the need to dedicate more time to the intervention compared to the control arm. All participants in the intervention group were also invited to participate in digital group meetings that, even though they were described as voluntary, could have put further pressure on the participants. Keeping all that in mind, an app tailored for the target group of young NEETs may contain shorter modules to not appear excessively demanding.

### Strengths and Limitations

Among the strengths of the study is the use of an RCT design using an active control group, which lay ground for an investigation of potential additional benefits of the specific app. The downside of having an active control group is that it makes it more difficult to detect effects [15,16,18]. Furthermore, the analyses made with both the PP and ITT approaches to handle missing data in the material due to high dropout resulted in similar results in all analyses. The power of the study was affected by the fact that recruitment had to close before reaching 180 participants due to logistical reasons. In addition, the dropout exceeded the expected 25%, which further lowered the power of the study. The online format also limited the possibilities to further explore the reasons for dropping out. Apart from a high dropout, there were also problems with low adherence to the intervention and low participation in the digital group meetings. About 1 in 10 participants were born outside of Sweden, which is about half the share compared to the Swedish setting, where 1 out of 5 in the age group is born outside of Sweden [57], and the numbers are too small in this sample to allow for assessing the effect of ethnicity which could have provided additional insights of the app’s effects. The recruitment strategy with potential participants volunteering to participate limits the generalizability of the findings due to the risk of self-selection bias. Another limitation is related to the lack of blinding in the trial, which could not be done due to the design of the study. Furthermore, the fact that limited qualitative data were collected limits the possibilities of providing explanations of the use and nonuse of the app or for potential reasons for dropping out.

### Conclusion

The findings of the study showed no differences in the effects of an app-based intervention for psychological well-being between young NEETs with disabilities and those without, although low adherence and high attrition make the conclusions weak. No effects were, however, seen between those using the app and those receiving film clips. The findings about a higher likelihood of being in employment or education among those who completed modules in the app compared to those who did not call for more research about the potential reasons. The study managed to reach a wide variety of young NEETs, reaching groups that often are hard to reach, such as participants with disabilities and young women. Many were also relatively new to the NEET situation and were not in contact with other activity initiatives, embedding for early interventions. There might be a need to include other social media sources for recruitment and to adjust the app and its content to increase the chances of including and retaining young men, since they were both underrepresented in the study population and overrepresented among those who dropped out. The heterogeneity of disabilities among the participants makes it difficult to conclude the findings, and more studies are needed to assess the appropriateness of digital interventions across diagnoses. NEETs with disabilities are of particular concern and might need additional efforts or other types of interventions than the one investigated in this study.

## Appendix

Multimedia Appendix 1 CONSORT-eHEALTH checklist (V 1.6.1).

Multimedia Appendix 2 Film clips to the control group.

Multimedia Appendix 3 Self reported health of participants at baseline.

Multimedia Appendix 4 Intervention group characteristics in relation to completion of modules in the app.

## Abbreviations

ACT: acceptance and commitment therapy
ADHD: attention-deficit/hyperactivity disorder
CONSORT-EHEALTH: Consolidated Standards of Reporting Trials of Electronic and Mobile Health Applications and Online Telehealth
GAD-7: Generalized Anxiety Disorder 7-item scale
GLM: general linear model
ITT: intention to treat
NEET: not in employment, education, or training
OECD: Organisation for Economic Co-operation and Development
PHQ-9: Patient Health Questionnaire-9
PP: per protocol
PSS-10: Perceived Stress Scale with 10 items
RCT: randomized controlled trial
REDCap: Research Electronic Data Capture
WHO-5: World Health Organization-Five Well-Being Index

## Data Availability

The datasets generated and/or analyzed during this study are not publicly available because the informed consent did not include a statement that the data would be made public. However, subsets of the data can be made available from the corresponding author on reasonable request and with permission from the Swedish Ethical Review Authority.
