# Supplementary material for: Effects of an App-Based Intervention on Psychological Well-Being Among Young Individuals not in Employment, Education, or Training With and Those Without Disability: Subgroup Analysis of a Randomized Controlled Trial
Source: JMIR Pediatr Parent. 2026 Feb 12;9:e71367. doi: 10.2196/71367 (PMC12946780; doi:10.2196/71367)
Supplement: Multimedia Appendix 1 [file pediatrics_v9i1e71367_app1.pdf]

CONSORT-EHEALTH (V 1.6.1) - Submission/Publication Form

The CONSORT-EHEALTH checklist is intended for authors of randomized trials evaluating web-based and Internet-based applications/interventions, including mobile interventions, electronic games (incl multiplayer games), social media, certain telehealth applications, and other interactive and/or networked electronic applications. Some of the items (e.g. all subitems under item 5 - description of the intervention) may also be applicable for other study designs.

The goal of the CONSORT EHEALTH checklist and guideline is to be
a) a guide for reporting for authors of RCTs,
b) to form a basis for appraisal of an ehealth trial (in terms of validity)

CONSORT-EHEALTH items/subitems are MANDATORY reporting items for studies published in the Journal of Medical Internet Research and other journals / scientific societies endorsing the checklist.

Items numbered 1., 2., 3., 4a., 4b etc are original CONSORT or CONSORT-NPT (non-pharmacologic treatment) items.
Items with Roman numerals (i., ii, iii, iv etc.) are CONSORT-EHEALTH extensions/clarifications.

As the CONSORT-EHEALTH checklist is still considered in a formative stage, we would ask that you also RATE ON A SCALE OF 1-5 how important/useful you feel each item is FOR THE PURPOSE OF THE CHECKLIST and reporting guideline (optional).

Mandatory reporting items are marked with a red *.
In the textboxes, either copy & paste the relevant sections from your manuscript into this form - please include any quotes from your manuscript in QUOTATION MARKS,
or answer directly by providing additional information not in the manuscript, or elaborating on why the item was not relevant for this study.

YOUR ANSWERS WILL BE PUBLISHED AS A SUPPLEMENTARY FILE TO YOUR PUBLICATION IN JMIR AND ARE CONSIDERED PART OF YOUR PUBLICATION (IF ACCEPTED).
Please fill in these questions diligently. Information will not be copyedited, so please use proper spelling and grammar, use correct capitalization, and avoid abbreviations.

DO NOT FORGET TO SAVE AS PDF _AND_ CLICK THE SUBMIT BUTTON SO YOUR ANSWERS ARE IN OUR DATABASE !!!

Citation Suggestion (if you append the pdf as Appendix we suggest to cite this paper in the caption):
Eysenbach G, CONSORT-EHEALTH Group.
[illegible]
J Med Internet Res 2011;13(4):e126

Du redigerar ditt svar. Om du delar den här webbadressen kan även andra redigera ditt svar.

FYLL I ETT NYTT SVAR

URL: http://www.jmir.org/2011/4/e126/
doi: 10.2196/jmir.1923
PMID: 22209829

lisa.blom@ki.se Byt konto

Inte delad

Skicka på nytt för att spara

* Anger obligatorisk fråga

Your name *

First Last

Lisa Blom

Primary Affiliation (short), City, Country *

University of Toronto, Toronto, Canada

Karolinska Institutet, Stockholm, Sweden

Your e-mail address *

abc@gmail.com

lisa.blom@ki.se

Title of your manuscript *

Provide the (draft) title of your manuscript.

Effects of an app-based intervention on psychological well-being among young individuals not in employment, education, or training with and without disability: a subgroup analysis of a randomized controlled trial

Du redigerar ditt svar. Om du delar den här webbadressen kan även andra redigera ditt svar.

FYLL I ETT NYTT SVAR

Name of your App/Software/Intervention *

If there is a short and a long/alternate name, write the short name first and add the long name in brackets.

29k is the name of the app

Evaluated Version (if any)

e.g. "V1", "Release 2017-03-01", "Version 2.0.27913"

Ditt svar

Language(s) *

What language is the intervention/app in? If multiple languages are available, separate by comma (e.g. "English, French")

English, Swedish

URL of your Intervention Website or App

e.g. a direct link to the mobile app on app in appstore (itunes, Google Play), or URL of the website. If the intervention is a DVD or hardware, you can also link to an Amazon page.

https://29k.org/

URL of an image/screenshot (optional)

Ditt svar

Du redigerar ditt svar. Om du delar den här webbadressen kan även andra redigera ditt svar.

FYLL I ETT NYTT SVAR

Accessibility *

Can an enduser access the intervention presently?

- (selected) access is free and open
- access only for special usergroups, not open
- access is open to everyone, but requires payment/subscription/in-app purchases
- app/intervention no longer accessible
- Övrigt:

Primary Medical Indication/Disease/Condition *

e.g. "Stress", "Diabetes", or define the target group in brackets after the condition, e.g. "Autism (Parents of children with)", "Alzheimers (Informal Caregivers of)"

Mental health, well-being and inner developme

Primary Outcomes measured in trial *

comma-separated list of primary outcomes reported in the trial

Well-being, Psychological distress

Secondary/other outcomes

Are there any other outcomes the intervention is expected to affect?

Activity level in terms of employment, studies or training

Du redigerar ditt svar. Om du delar den här webbadressen kan även andra redigera ditt svar.

FYLL I ETT NYTT SVAR

Recommended "Dose" *

What do the instructions for users say on how often the app should be used?

- ◯ Approximately Daily
- ◉ Approximately Weekly
- ◯ Approximately Monthly
- ◯ Approximately Yearly
- ◯ "as needed"
- ◯ Övrigt:

Approx. Percentage of Users (starters) still using the app as recommended after 3 months *

- ◉ unknown / not evaluated
- ◯ 0-10%
- ◯ 11-20%
- ◯ 21-30%
- ◯ 31-40%
- ◯ 41-50%
- ◯ 51-60%
- ◯ 61-70%
- ◯ 71%-80%
- ◯ 81-90%
- ◯ 91-100%
- ◯ Övrigt:

Du redigerar ditt svar. Om du delar den här webbadressen kan även andra redigera ditt svar.

FYLL I ETT NYTT SVAR

Overall, was the app/intervention effective? *

- ( ) yes: all primary outcomes were significantly better in intervention group vs control
- ( ) partly: SOME primary outcomes were significantly better in intervention group vs control
- (•) no statistically significant difference between control and intervention
- ( ) potentially harmful: control was significantly better than intervention in one or more outcomes
- ( ) inconclusive: more research is needed
- ( ) Övrigt:

Article Preparation Status/Stage *

At which stage in your article preparation are you currently (at the time you fill in this form)

- ( ) not submitted yet - in early draft status
- (•) not submitted yet - in late draft status, just before submission
- ( ) submitted to a journal but not reviewed yet
- ( ) submitted to a journal and after receiving initial reviewer comments
- ( ) submitted to a journal and accepted, but not published yet
- ( ) published
- ( ) Övrigt:

Du redigerar ditt svar. Om du delar den här webbadressen kan även andra redigera ditt svar.

FYLL I ETT NYTT SVAR

Journal *

If you already know where you will submit this paper (or if it is already submitted), please provide the journal name (if it is not JMIR, provide the journal name under "other")

- (selected) not submitted yet / unclear where I will submit this
- Journal of Medical Internet Research (JMIR)
- JMIR mHealth and UHealth
- JMIR Serious Games
- JMIR Mental Health
- JMIR Public Health
- JMIR Formative Research
- Other JMIR sister journal
- Övrigt:

Is this a full powered effectiveness trial or a pilot/feasibility trial? *

- Pilot/feasibility
- (selected) Fully powered

Manuscript tracking number *

If this is a JMIR submission, please provide the manuscript tracking number under "other" (The ms tracking number can be found in the submission acknowledgement email, or when you login as author in JMIR. If the paper is already published in JMIR, then the ms tracking number is the four-digit number at the end of the DOI, to be found at the bottom of each published article in JMIR)

- (selected) no ms number (yet) / not (yet) submitted to / published in JMIR
- Övrigt:

Du redigerar ditt svar. Om du delar den här webbadressen kan även andra redigera ditt svar.

FYLL I ETT NYTT SVAR

TITLE AND ABSTRACT

1a) TITLE: Identification as a randomized trial in the title

1a) Does your paper address CONSORT item 1a? *

I.e does the title contain the phrase "Randomized Controlled Trial"? (if not, explain the reason under "other")

- (x) yes
- ( ) Övrigt:

1a-i) Identify the mode of delivery in the title

Identify the mode of delivery. Preferably use “web-based” and/or “mobile” and/or “electronic game” in the title. Avoid ambiguous terms like “online”, “virtual”, “interactive”. Use “Internet-based” only if Intervention includes non-web-based Internet components (e.g. email), use “computer-based” or “electronic” only if offline products are used. Use “virtual” only in the context of “virtual reality” (3-D worlds). Use “online” only in the context of “online support groups”. Complement or substitute product names with broader terms for the class of products (such as “mobile” or “smart phone” instead of “iphone”), especially if the application runs on different platforms.

	1	2	3	4	5	
subitem not at all important	( )	( )	( )	( )	(x)	essential

Rensa markering

Does your paper address subitem 1a-i? *

Copy and paste relevant sections from manuscript title (include quotes in quotation marks "like this" to indicate direct quotes from your manuscript), or elaborate on this item by providing additional information not in the ms, or briefly explain why the item is not applicable/relevant for your study

Du redigerar ditt svar. Om du delar den här webbadressen kan även andra redigera ditt svar.

FYLL I ETT NYTT SVAR

1a-ii) Non-web-based components or important co-interventions in title

Mention non-web-based components or important co-interventions in title, if any (e.g., “with telephone support”).

	1	2	3	4	5	
subitem not at all important	○	○	●	○	○	essential

Rensa markering

Does your paper address subitem 1a-ii?

Copy and paste relevant sections from manuscript title (include quotes in quotation marks "like this" to indicate direct quotes from your manuscript), or elaborate on this item by providing additional information not in the ms, or briefly explain why the item is not applicable/relevant for your study

The intervention group are also invited to group meetings but that was voluntary to participate in and it is not included in the title

1a-iii) Primary condition or target group in the title

Mention primary condition or target group in the title, if any (e.g., “for children with Type I Diabetes”) Example: A Web-based and Mobile Intervention with Telephone Support for Children with Type I Diabetes: Randomized Controlled Trial

	1	2	3	4	5	
subitem not at all important	○	○	○	○	●	essential

Rensa markering

Du redigerar ditt svar. Om du delar den här webbadressen kan även andra redigera ditt svar.

FYLL I ETT NYTT SVAR

Does your paper address subitem 1a-iii? *

Copy and paste relevant sections from manuscript title (include quotes in quotation marks "like this" to indicate direct quotes from your manuscript), or elaborate on this item by providing additional information not in the ms, or briefly explain why the item is not applicable/relevant for your study

We state "psychological well-being among young individuals not in employment, education, or training with and without disability"

1b) ABSTRACT: Structured summary of trial design, methods, results, and conclusions

NPT extension: Description of experimental treatment, comparator, care providers, centers, and blinding status.

1b-i) Key features/functionalities/components of the intervention and comparator in the METHODS section of the ABSTRACT

Mention key features/functionalities/components of the intervention and comparator in the abstract. If possible, also mention theories and principles used for designing the site. Keep in mind the needs of systematic reviewers and indexers by including important synonyms. (Note: Only report in the abstract what the main paper is reporting. If this information is missing from the main body of text, consider adding it)

	1	2	3	4	5	
subitem not at all important	○	○	○	○	◉	essential

Rensa markering

Does your paper address subitem 1b-i? *

Copy and paste relevant sections from the manuscript abstract (include quotes in quotation marks "like this" to indicate direct quotes from your manuscript), or elaborate on this item by providing additional information not in the ms, or briefly explain why the item is not applicable/relevant for your study

"The intervention group (n=77) used an app for psychological well-being with possibility for digital group meetings for 6 weeks and the control group (n=74) received film clips once a

Du redigerar ditt svar. Om du delar den här webbadressen kan även andra redigera ditt svar.

FYLL I ETT NYTT SVAR

1b-ii) Level of human involvement in the METHODS section of the ABSTRACT

Clarify the level of human involvement in the abstract, e.g., use phrases like “fully automated” vs. “therapist/nurse/care provider/physician-assisted” (mention number and expertise of providers involved, if any). (Note: Only report in the abstract what the main paper is reporting. If this information is missing from the main body of text, consider adding it)

	1	2	3	4	5	
subitem not at all important	○	○	○	◉	○	essential

Rensa markering

Does your paper address subitem 1b-ii?

Copy and paste relevant sections from the manuscript abstract (include quotes in quotation marks "like this" to indicate direct quotes from your manuscript), or elaborate on this item by providing additional information not in the ms, or briefly explain why the item is not applicable/relevant for your study

"an app for psychological well-being with possibility for digital group meetings"

1b-iii) Open vs. closed, web-based (self-assessment) vs. face-to-face assessments in the METHODS section of the ABSTRACT

Mention how participants were recruited (online vs. offline), e.g., from an open access website or from a clinic or a closed online user group (closed usergroup trial), and clarify if this was a purely web-based trial, or there were face-to-face components (as part of the intervention or for assessment). Clearly say if outcomes were self-assessed through questionnaires (as common in web-based trials). Note: In traditional offline trials, an open trial (open-label trial) is a type of clinical trial in which both the researchers and participants know which treatment is being administered. To avoid confusion, use “blinded” or “unblinded” to indicated the level of blinding instead of “open”, as “open” in web-based trials usually refers to “open access” (i.e. participants can self-enrol). (Note: Only report in the abstract what the main paper is reporting. If this information is missing from the main body of text, consider adding it)

	1	2	3	4	5	
subitem not at all important	○	○	○	○	◉	essential

Du redigerar ditt svar. Om du delar den här webbadressen kan även andra redigera ditt svar.

FYLL I ETT NYTT SVAR

Does your paper address subitem 1b-iii?

Copy and paste relevant sections from the manuscript abstract (include quotes in quotation marks "like this" to indicate direct quotes from your manuscript), or elaborate on this item by providing additional information not in the ms, or briefly explain why the item is not applicable/relevant for your study

"Participants were recruited mainly via social media platforms and through organizations working with NEET individuals." and "Outcomes were self-assessed through questionnaires."

1b-iv) RESULTS section in abstract must contain use data

Report number of participants enrolled/assessed in each group, the use/uptake of the intervention (e.g., attrition/adherence metrics, use over time, number of logins etc.), in addition to primary/secondary outcomes. (Note: Only report in the abstract what the main paper is reporting. If this information is missing from the main body of text, consider adding it)

	1	2	3	4	5	
subitem not at all important	○	○	○	●	○	essential

Rensa markering

Does your paper address subitem 1b-iv?

Copy and paste relevant sections from the manuscript abstract (include quotes in quotation marks "like this" to indicate direct quotes from your manuscript), or elaborate on this item by providing additional information not in the ms, or briefly explain why the item is not applicable/relevant for your study

"Usage data show that 68.6% of the participants in the intervention group downloaded the app and 24.7% completed all six modules. "

Du redigerar ditt svar. Om du delar den här webbadressen kan även andra redigera ditt svar.

FYLL I ETT NYTT SVAR

1b-v) CONCLUSIONS/DISCUSSION in abstract for negative trials

Conclusions/Discussions in abstract for negative trials: Discuss the primary outcome - if the trial is negative (primary outcome not changed), and the intervention was not used, discuss whether negative results are attributable to lack of uptake and discuss reasons. (Note: Only report in the abstract what the main paper is reporting. If this information is missing from the main body of text, consider adding it)

	1	2	3	4	5	
subitem not at all important	○	○	○	○	◉	essential

Rensa markering

Does your paper address subitem 1b-v?

Copy and paste relevant sections from the manuscript abstract (include quotes in quotation marks "like this" to indicate direct quotes from your manuscript), or elaborate on this item by providing additional information not in the ms, or briefly explain why the item is not applicable/relevant for your study

"NEETs with disability are of particular concern and might need additional efforts or other types of interventions than the one investigated herein. Findings can be considered weak due to the low adherence and high attrition."

INTRODUCTION

2a) In INTRODUCTION: Scientific background and explanation of rationale

Du redigerar ditt svar. Om du delar den här webbadressen kan även andra redigera ditt svar.

FYLL I ETT NYTT SVAR

2a-i) Problem and the type of system/solution

Describe the problem and the type of system/solution that is object of the study: intended as stand-alone intervention vs. incorporated in broader health care program? Intended for a particular patient population? Goals of the intervention, e.g., being more cost-effective to other interventions, replace or complement other solutions? (Note: Details about the intervention are provided in “Methods” under 5)

	1	2	3	4	5	
subitem not at all important	○	○	○	○	◉	essential

Rensa markering

Does your paper address subitem 2a-i? *

Copy and paste relevant sections from the manuscript (include quotes in quotation marks "like this" to indicate direct quotes from your manuscript), or elaborate on this item by providing additional information not in the ms, or briefly explain why the item is not applicable/relevant for your study

"In line with the research gaps identified relating to differences in how subgroups of NEETs make use of, and gain effects from, mental health interventions, the study aimed to expand the knowledge on the effects of an app-based intervention built on acceptance and commitment therapy on NEETs with and without disability. "

2a-ii) Scientific background, rationale: What is known about the (type of) system

Scientific background, rationale: What is known about the (type of) system that is the object of the study (be sure to discuss the use of similar systems for other conditions/diagnoses, if appropiate), motivation for the study, i.e. what are the reasons for and what is the context for this specific study, from which stakeholder viewpoint is the study performed, potential impact of findings [2]. Briefly justify the choice of the comparator.

	1	2	3	4	5	
subitem not at all important	○	○	○	○	◉	essential

Rensa markering

Du redigerar ditt svar. Om du delar den här webbadressen kan även andra redigera ditt svar.

FYLL I ETT NYTT SVAR

Does your paper address subitem 2a-ii? *

Copy and paste relevant sections from the manuscript (include quotes in quotation marks "like this" to indicate direct quotes from your manuscript), or elaborate on this item by providing additional information not in the ms, or briefly explain why the item is not applicable/relevant for your study

"Web-based acceptance and commitment therapy has showed promising results in reducing stress and increase academic buoyancy among adolescents (Puolakanaho et al., 2019) and improving psychological, emotional and social wellbeing, life satisfaction and self-esteem and reducing symptoms of stress and depression among university students (Rasanen, Lappalainen, Muotka, Tolvanen, & Lappalainen, 2016). "

2b) In INTRODUCTION: Specific objectives or hypotheses

Does your paper address CONSORT subitem 2b? *

Copy and paste relevant sections from the manuscript (include quotes in quotation marks "like this" to indicate direct quotes from your manuscript), or elaborate on this item by providing additional information not in the ms, or briefly explain why the item is not applicable/relevant for your study

We have included aim and research questions: ", the study aimed to expand the knowledge on the effects of an app-based intervention built on acceptance and commitment therapy on NEETs with and without disability. The following research question was considered: How do the effects (well-being, psychological distress and activity level) of an app-based intervention on psychological well-being on young NEETs in Sweden vary depending on presence of disability and moderated by background characteristics? "

METHODS

3a) Description of trial design (such as parallel, factorial) including allocation ratio

Du redigerar ditt svar. Om du delar den här webbadressen kan även andra redigera ditt svar.

FYLL I ETT NYTT SVAR

Does your paper address CONSORT subitem 3a? *

Copy and paste relevant sections from the manuscript (include quotes in quotation marks "like this" to indicate direct quotes from your manuscript), or elaborate on this item by providing additional information not in the ms, or briefly explain why the item is not applicable/relevant for your study

"A two-armed parallel randomized controlled trial (RCT) was conducted between March to October 2021 to assess feasibility and effectiveness of an app-based intervention built on Acceptance and Commitment Therapy (ACT) in comparison to film clips from YouTube dealing with mental health problems. " and "Randomization was performed 1:1"

3b) Important changes to methods after trial commencement (such as eligibility criteria), with reasons

Does your paper address CONSORT subitem 3b? *

Copy and paste relevant sections from the manuscript (include quotes in quotation marks "like this" to indicate direct quotes from your manuscript), or elaborate on this item by providing additional information not in the ms, or briefly explain why the item is not applicable/relevant for your study

"The recruitment of participants from all over Sweden was done in two different phases, with the first phase of the recruitment taking place in March and April 2021 with advertisements on Facebook and Instagram. At this stage, municipalities and organizations working with NEETs were contacted and asked for support to reach out by posting on their social medias, informing about the study to potential participants or having the study coordinator inform at digital group meetings. The study was also advertised on the Karolinska Institutet website “Research subjects wanted”. When a participant declared interest to participate in the study at this stage, s/he received an email with a link to a participant information and consent form, followed by a baseline questionnaire. Despite the efforts made to reach out broadly to the target group, recruitment went slow, which is why a new take on the recruitment strategy had to be made. Phase two of the recruitment started in May 2021 when the developer of the app that was tested in the study helped to create a short recruitment video that could be posted on other social media sites such as Snapchat, TikTok and YouTube. Snapchat became the primary recruitment source. Those who declared interest at this phase were sent a text message where they were asked if the study coordinator could phone them to give them more information about the study or if they preferred to be emailed. "

Du redigerar ditt svar. Om du delar den här webbadressen kan även andra redigera ditt svar.

FYLL I ETT NYTT SVAR

3b-i) Bug fixes, Downtimes, Content Changes

Bug fixes, Downtimes, Content Changes: ehealth systems are often dynamic systems. A description of changes to methods therefore also includes important changes made on the intervention or comparator during the trial (e.g., major bug fixes or changes in the functionality or content) (5-iii) and other “unexpected events” that may have influenced study design such as staff changes, system failures/downtimes, etc. [2].

	1	2	3	4	5	
subitem not at all important	○	○	●	○	○	essential

Rensa markering

Does your paper address subitem 3b-i?

Copy and paste relevant sections from the manuscript (include quotes in quotation marks "like this" to indicate direct quotes from your manuscript), or elaborate on this item by providing additional information not in the ms, or briefly explain why the item is not applicable/relevant for your study

No bug fixes or content changes were necessary during the trial.

4a) Eligibility criteria for participants

Du redigerar ditt svar. Om du delar den här webbadressen kan även andra redigera ditt svar.

FYLL I ETT NYTT SVAR

Does your paper address CONSORT subitem 4a? *

Copy and paste relevant sections from the manuscript (include quotes in quotation marks "like this" to indicate direct quotes from your manuscript), or elaborate on this item by providing additional information not in the ms, or briefly explain why the item is not applicable/relevant for your study

"To be eligible for participation in the study, participants had to be 16-24 years of age, unemployed or not working or studying more than 20 hours per week. They also had to be willing and have the possibility to participate in the full intervention and have sufficient level of Swedish to take part in the intervention. Participants who received high values on screening for depression, i.e., 15 points or more on the Patient Health Questionnaire (PHQ9) were excluded and provided contact details on where to seek care. The reason for exclusion was that depression was considered to need more treatment than this study intervention could provide. The excluded participants were also offered the chance to be contacted by any of the psychologists involved in the study if they wished to have further support. "

4a-i) Computer / Internet literacy

Computer / Internet literacy is often an implicit “de facto” eligibility criterion - this should be explicitly clarified.

	1	2	3	4	5	
subitem not at all important	○	○	◉	○	○	essential

Rensa markering

Does your paper address subitem 4a-i?

Copy and paste relevant sections from the manuscript (include quotes in quotation marks "like this" to indicate direct quotes from your manuscript), or elaborate on this item by providing additional information not in the ms, or briefly explain why the item is not applicable/relevant for your study

We do not discuss internet literacy in the manuscript since we expect the target group of the study to have an overall high internet literacy.

Du redigerar ditt svar. Om du delar den här webbadressen kan även andra redigera ditt svar.

FYLL I ETT NYTT SVAR

4a-ii) Open vs. closed, web-based vs. face-to-face assessments:

Open vs. closed, web-based vs. face-to-face assessments: Mention how participants were recruited (online vs. offline), e.g., from an open access website or from a clinic, and clarify if this was a purely web-based trial, or there were face-to-face components (as part of the intervention or for assessment), i.e., to what degree got the study team to know the participant. In online-only trials, clarify if participants were quasi-anonymous and whether having multiple identities was possible or whether technical or logistical measures (e.g., cookies, email confirmation, phone calls) were used to detect/prevent these.

	1	2	3	4	5	
subitem not at all important	○	○	○	○	◉	essential

Rensa markering

Does your paper address subitem 4a-ii? *

Copy and paste relevant sections from the manuscript (include quotes in quotation marks "like this" to indicate direct quotes from your manuscript), or elaborate on this item by providing additional information not in the ms, or briefly explain why the item is not applicable/relevant for your study

Recruitment of participants took place online but where we also had contact with organizations and municipalities that are working with needs, this information is described in the section "Recruitment". In the same section we also describe how the participants were contacted when filing interest.

4a-iii) Information giving during recruitment

Information given during recruitment. Specify how participants were briefed for recruitment and in the informed consent procedures (e.g., publish the informed consent documentation as appendix, see also item X26), as this information may have an effect on user self-selection, user expectation and may also bias results.

	1	2	3	4	5	
subitem not at all important	○	○	○	○	◉	essential

Rensa markering

Du redigerar ditt svar. Om du delar den här webbadressen kan även andra redigera ditt svar.

FYLL I ETT NYTT SVAR

Does your paper address subitem 4a-iii?

Copy and paste relevant sections from the manuscript (include quotes in quotation marks "like this" to indicate direct quotes from your manuscript), or elaborate on this item by providing additional information not in the ms, or briefly explain why the item is not applicable/relevant for your study

"When a participant declared interest to participate in the study at this stage, s/he received an email with a link to a participant information and consent form, followed by a baseline questionnaire. " AND "Those who declared interest at this phase were sent a text message where they were asked if the study coordinator could phone them to give them more information about the study or if they preferred to be emailed. "

4b) Settings and locations where the data were collected

Does your paper address CONSORT subitem 4b? *

Copy and paste relevant sections from the manuscript (include quotes in quotation marks "like this" to indicate direct quotes from your manuscript), or elaborate on this item by providing additional information not in the ms, or briefly explain why the item is not applicable/relevant for your study

"The recruitment of participants from all over Sweden was done in two different phases, with the first phase of the recruitment taking place in March and April 2021 with advertisements on Facebook and Instagram."

4b-i) Report if outcomes were (self-)assessed through online questionnaires

Clearly report if outcomes were (self-)assessed through online questionnaires (as common in web-based trials) or otherwise.

	1	2	3	4	5	
subitem not at all important	○	○	○	○	◉	essential

Rensa markering

Du redigerar ditt svar. Om du delar den här webbadressen kan även andra redigera ditt svar.

FYLL I ETT NYTT SVAR

Does your paper address subitem 4b-i? *

Copy and paste relevant sections from the manuscript (include quotes in quotation marks "like this" to indicate direct quotes from your manuscript), or elaborate on this item by providing additional information not in the ms, or briefly explain why the item is not applicable/relevant for your study

"Data were collected using self-assessed electronically distributed questionnaires at three timepoints: baseline (t1), post-intervention (t2), and six months after randomization (t3). "

4b-ii) Report how institutional affiliations are displayed

Report how institutional affiliations are displayed to potential participants [on ehealth media], as affiliations with prestigious hospitals or universities may affect volunteer rates, use, and reactions with regards to an intervention.(Not a required item – describe only if this may bias results)

	1	2	3	4	5	
subitem not at all important	○	○	○	○	◉	essential

Rensa markering

Does your paper address subitem 4b-ii?

Copy and paste relevant sections from the manuscript (include quotes in quotation marks "like this" to indicate direct quotes from your manuscript), or elaborate on this item by providing additional information not in the ms, or briefly explain why the item is not applicable/relevant for your study

Ditt svar

5) The interventions for each group with sufficient details to allow replication, including how and when they were actually administered

Du redigerar ditt svar. Om du delar den här webbadressen kan även andra redigera ditt svar.

FYLL I ETT NYTT SVAR

5-i) Mention names, credential, affiliations of the developers, sponsors, and owners

Mention names, credential, affiliations of the developers, sponsors, and owners [6] (if authors/evaluators are owners or developer of the software, this needs to be declared in a "Conflict of interest" section or mentioned elsewhere in the manuscript).

	1	2	3	4	5	
subitem not at all important	○	○	○	○	◉	essential

Rensa markering

Does your paper address subitem 5-i?

Copy and paste relevant sections from the manuscript (include quotes in quotation marks "like this" to indicate direct quotes from your manuscript), or elaborate on this item by providing additional information not in the ms, or briefly explain why the item is not applicable/relevant for your study

"FL and JR have positions both at Karolinska Institutet and at the non-profitable foundation 29k that developed the app. Both FL and JR have participated in the development and the content of the platform 29k as employed at 29k. Neither FL or JR were involved in the analysis of the data and the foundation 29k have not been involved either in the design of the study or in the manuscript writing. LB and LL were in charge of the analysis and have no competing interests." AND "Last but not least, we would like to thank the Swedish Research Council for Health, Working Life and Welfare (Forte) who funded the study (2015-01136). The funders had no role in the design of the study or the reporting of the results."

5-ii) Describe the history/development process

Describe the history/development process of the application and previous formative evaluations (e.g., focus groups, usability testing), as these will have an impact on adoption/use rates and help with interpreting results.

	1	2	3	4	5	
subitem not at all important	○	○	◉	○	○	essential

Rensa markering

Du redigerar ditt svar. Om du delar den här webbadressen kan även andra redigera ditt svar.

FYLL I ETT NYTT SVAR

Does your paper address subitem 5-ii?

Copy and paste relevant sections from the manuscript (include quotes in quotation marks "like this" to indicate direct quotes from your manuscript), or elaborate on this item by providing additional information not in the ms, or briefly explain why the item is not applicable/relevant for your study

We have not included information about the development process but written the following: "The 29k app was developed by the non-profit foundation 29k with the objective to make evidence-based tools for mental health available for as many as possible in a user-friendly manner. The youth version used in the study was in Swedish and consisted of six modules that were to be used during six weeks. "

5-iii) Revisions and updating

Revisions and updating. Clearly mention the date and/or version number of the application/intervention (and comparator, if applicable) evaluated, or describe whether the intervention underwent major changes during the evaluation process, or whether the development and/or content was “frozen” during the trial. Describe dynamic components such as news feeds or changing content which may have an impact on the replicability of the intervention (for unexpected events see item 3b).

	1	2	3	4	5	
subitem not at all important	○	○	○	◉	○	essential

Rensa markering

Does your paper address subitem 5-iii?

Copy and paste relevant sections from the manuscript (include quotes in quotation marks "like this" to indicate direct quotes from your manuscript), or elaborate on this item by providing additional information not in the ms, or briefly explain why the item is not applicable/relevant for your study

"The modules included interactive material and exercises on stress, self-compassion, gratitude, relationship skills, recovery and mindfulness exercises. In the app additional modules were freely available for the participants to use but no updates of the app were made why the content remained the same throughout the trial. "

Du redigerar ditt svar. Om du delar den här webbadressen kan även andra redigera ditt svar.

FYLL I ETT NYTT SVAR

5-iv) Quality assurance methods

Provide information on quality assurance methods to ensure accuracy and quality of information provided [1], if applicable.

	1	2	3	4	5	
subitem not at all important	○	○	○	◉	○	essential

Rensa markering

Does your paper address subitem 5-iv?

Copy and paste relevant sections from the manuscript (include quotes in quotation marks "like this" to indicate direct quotes from your manuscript), or elaborate on this item by providing additional information not in the ms, or briefly explain why the item is not applicable/relevant for your study

A check of treatment information was not performed as the interventions offered were structured with standardized feedback to the intervention group. Regular supervision by a clinical psychologist was offered to group leaders.

5-v) Ensure replicability by publishing the source code, and/or providing screenshots/screen-capture video, and/or providing flowcharts of the algorithms used

Ensure replicability by publishing the source code, and/or providing screenshots/screen-capture video, and/or providing flowcharts of the algorithms used. Replicability (i.e., other researchers should in principle be able to replicate the study) is a hallmark of scientific reporting.

	1	2	3	4	5	
subitem not at all important	○	○	◉	○	○	essential

Rensa markering

Du redigerar ditt svar. Om du delar den här webbadressen kan även andra redigera ditt svar.

FYLL I ETT NYTT SVAR

Does your paper address subitem 5-v?

Copy and paste relevant sections from the manuscript (include quotes in quotation marks "like this" to indicate direct quotes from your manuscript), or elaborate on this item by providing additional information not in the ms, or briefly explain why the item is not applicable/relevant for your study

No screenshots are made available but a link to the website of the app. Links to the film clips sent to the control group are available in supplementary file. Source codes included in the data analysis are available upon request to the corresponding author.

5-vi) Digital preservation

Digital preservation: Provide the URL of the application, but as the intervention is likely to change or disappear over the course of the years; also make sure the intervention is archived (Internet Archive, webcitation.org, and/or publishing the source code or screenshots/videos alongside the article). As pages behind login screens cannot be archived, consider creating demo pages which are accessible without login.

	1	2	3	4	5	
subitem not at all important	○	○	○	●	○	essential

Rensa markering

Does your paper address subitem 5-vi?

Copy and paste relevant sections from the manuscript (include quotes in quotation marks "like this" to indicate direct quotes from your manuscript), or elaborate on this item by providing additional information not in the ms, or briefly explain why the item is not applicable/relevant for your study

We have not addressed this issue as content from the intervention platform (www.29k.org) still is available.

Du redigerar ditt svar. Om du delar den här webbadressen kan även andra redigera ditt svar.

FYLL I ETT NYTT SVAR

5-vii) Access

Access: Describe how participants accessed the application, in what setting/context, if they had to pay (or were paid) or not, whether they had to be a member of specific group. If known, describe how participants obtained “access to the platform and Internet” [1]. To ensure access for editors/reviewers/readers, consider to provide a “backdoor” login account or demo mode for reviewers/readers to explore the application (also important for archiving purposes, see vi).

	1	2	3	4	5	
subitem not at all important	○	○	○	●	○	essential

Rensa markering

Does your paper address subitem 5-vii? *

Copy and paste relevant sections from the manuscript (include quotes in quotation marks "like this" to indicate direct quotes from your manuscript), or elaborate on this item by providing additional information not in the ms, or briefly explain why the item is not applicable/relevant for your study

"Participants randomized to the intervention group received individual unique links to download the app. "

Du redigerar ditt svar. Om du delar den här webbadressen kan även andra redigera ditt svar.

FYLL I ETT NYTT SVAR

5-viii) Mode of delivery, features/functionalities/components of the intervention and comparator, and the theoretical framework

Describe mode of delivery, features/functionalities/components of the intervention and comparator, and the theoretical framework [6] used to design them (instructional strategy [1], behaviour change techniques, persuasive features, etc., see e.g., [7, 8] for terminology). This includes an in-depth description of the content (including where it is coming from and who developed it) [1],” whether [and how] it is tailored to individual circumstances and allows users to track their progress and receive feedback” [6]. This also includes a description of communication delivery channels and – if computer-mediated communication is a component – whether communication was synchronous or asynchronous [6]. It also includes information on presentation strategies [1], including page design principles, average amount of text on pages, presence of hyperlinks to other resources, etc. [1].

	1	2	3	4	5	
subitem not at all important	○	○	○	○	◉	essential

Rensa markering

Du redigerar ditt svar. Om du delar den här webbadressen kan även andra redigera ditt svar.

FYLL I ETT NYTT SVAR

Does your paper address subitem 5-viii? *

Copy and paste relevant sections from the manuscript (include quotes in quotation marks "like this" to indicate direct quotes from your manuscript), or elaborate on this item by providing additional information not in the ms, or briefly explain why the item is not applicable/relevant for your study

"The youth version used in the study was in Swedish and consisted of six modules that were to be used during six weeks. The modules included interactive material and exercises on stress, self-compassion, gratitude, relationship skills, recovery and mindfulness exercises. In the app additional modules were freely available for the participants to use but no updates of the app were made why the content remained the same throughout the trial. Participants randomized to the intervention group received individual unique links to download the app. Participants in the intervention group were invited to participate in weekly digital group meetings led by a trained facilitator. The facilitators did not need to be mental health professionals and got 1.5 hours of training in how to facilitate the groups. The meetings were 45 minutes long. Participation in the digital group meetings was voluntary and the participants could choose to participate in one or several or decide to use the app program by themselves without participating in the meetings. Reminders were emailed for group meetings and also worked as reminders for using the app." AND "The control intervention consisted of YouTube film clips where young adults were discussing stress, mental health problems, self-esteem, and the meaning of life. Link to the film clips were sent out via email once a week for six weeks and the clips were between 4-8 minutes long. The participants were asked to watch the video that was provided in the link and reflect for themselves. A reminder was sent out three days after the link was sent."

5-ix) Describe use parameters

Describe use parameters (e.g., intended "doses" and optimal timing for use). Clarify what instructions or recommendations were given to the user, e.g., regarding timing, frequency, heaviness of use, if any, or was the intervention used ad libitum.

	1	2	3	4	5	
subitem not at all important	○	○	○	○	◉	essential

Rensa markering

Du redigerar ditt svar. Om du delar den här webbadressen kan även andra redigera ditt svar.

FYLL I ETT NYTT SVAR

Does your paper address subitem 5-ix?

Copy and paste relevant sections from the manuscript (include quotes in quotation marks "like this" to indicate direct quotes from your manuscript), or elaborate on this item by providing additional information not in the ms, or briefly explain why the item is not applicable/relevant for your study

" The meetings were 45 minutes long. Participation in the digital group meetings was voluntary and the participants could choose to participate in one or several or decide to use the app program on an at least weekly basis by themselves without participating in the meetings. " AND "Link to the film clips were sent out via email once a week for six weeks and the clips were between 4-8 minutes long. The participants were asked to watch the video that was provided in the link and reflect for themselves. "

5-x) Clarify the level of human involvement

Clarify the level of human involvement (care providers or health professionals, also technical assistance) in the e-intervention or as co-intervention (detail number and expertise of professionals involved, if any, as well as “type of assistance offered, the timing and frequency of the support, how it is initiated, and the medium by which the assistance is delivered”. It may be necessary to distinguish between the level of human involvement required for the trial, and the level of human involvement required for a routine application outside of a RCT setting (discuss under item 21 – generalizability).

	1	2	3	4	5	
subitem not at all important	○	○	○	◉	○	essential

Rensa markering

Du redigerar ditt svar. Om du delar den här webbadressen kan även andra redigera ditt svar.

FYLL I ETT NYTT SVAR

Does your paper address subitem 5-x?

Copy and paste relevant sections from the manuscript (include quotes in quotation marks "like this" to indicate direct quotes from your manuscript), or elaborate on this item by providing additional information not in the ms, or briefly explain why the item is not applicable/relevant for your study

"Participants in the intervention group were invited to participate in weekly digital group meetings led by a trained facilitator. The facilitators did not need to be mental health professionals and got 1.5 hours of training in how to facilitate the groups. The meetings were 45 minutes long. Participation in the digital group meetings was voluntary and the participants could choose to participate in one or several or decide to use the app program on an at least weekly basis by themselves without participating in the meetings." AND "The participants were asked to watch the video that was provided in the link and reflect for themselves."

5-xi) Report any prompts/reminders used

Report any prompts/reminders used: Clarify if there were prompts (letters, emails, phone calls, SMS) to use the application, what triggered them, frequency etc. It may be necessary to distinguish between the level of prompts/reminders required for the trial, and the level of prompts/reminders for a routine application outside of a RCT setting (discuss under item 21 – generalizability).

	1	2	3	4	5	
subitem not at all important	○	○	○	○	◉	essential

Rensa markering

Does your paper address subitem 5-xi? *

Copy and paste relevant sections from the manuscript (include quotes in quotation marks "like this" to indicate direct quotes from your manuscript), or elaborate on this item by providing additional information not in the ms, or briefly explain why the item is not applicable/relevant for your study

"Reminders were emailed for group meetings and also worked as reminders for using the app." AND for the control group: "A reminder was sent out three days after the link was sent."

Du redigerar ditt svar. Om du delar den här webbadressen kan även andra redigera ditt svar.

FYLL I ETT NYTT SVAR

5-xii) Describe any co-interventions (incl. training/support)

Describe any co-interventions (incl. training/support): Clearly state any interventions that are provided in addition to the targeted eHealth intervention, as ehealth intervention may not be designed as stand-alone intervention. This includes training sessions and support [1]. It may be necessary to distinguish between the level of training required for the trial, and the level of training for a routine application outside of a RCT setting (discuss under item 21 – generalizability.

	1	2	3	4	5	
subitem not at all important	○	○	○	○	◉	essential

Rensa markering

Does your paper address subitem 5-xii? *

Copy and paste relevant sections from the manuscript (include quotes in quotation marks "like this" to indicate direct quotes from your manuscript), or elaborate on this item by providing additional information not in the ms, or briefly explain why the item is not applicable/relevant for your study

"Participants in the intervention group were invited to participate in weekly digital group meetings led by a trained facilitator. The facilitators did not need to be mental health professionals and got 1.5 hours of training in how to facilitate the groups. The meetings were 45 minutes long. Participation in the digital group meetings was voluntary and the participants could choose to participate in one or several or decide to use the app program on an at least weekly basis by themselves without participating in the meetings."

6a) Completely defined pre-specified primary and secondary outcome measures, including how and when they were assessed

Du redigerar ditt svar. Om du delar den här webbadressen kan även andra redigera ditt svar.

FYLL I ETT NYTT SVAR

Does your paper address CONSORT subitem 6a? *

Copy and paste relevant sections from the manuscript (include quotes in quotation marks "like this" to indicate direct quotes from your manuscript), or elaborate on this item by providing additional information not in the ms, or briefly explain why the item is not applicable/relevant for your study

"Data were collected using self-assessed electronically distributed questionnaires at three timepoints: baseline (t1), post-intervention (t2), and six months after randomization (t3). The secure web platform RedCap was used for overall project management and for data collection by sending out links to the questionnaires as well as reminders when the questionnaires had not been completed. The questionnaire included background questions on age, sex, country of birth, physical or psychological disability or diagnose, years of schooling, work experience, relationship with parents, trust in different societal institutions, trust in general and how they found out about the study. The following outcome measures were included in the questionnaire:Self-esteem. Self-esteem was measured with the Swedish version of the Rosenberg self-esteem scale (Rosenberg, 1965) consisting of 10 items to be answered with a 4-grade scale stretching from “strongly agree” to “strongly disagree”. Well-being. The Swedish version of the World Health Organization-Five Well-Being Index (WHO-5) (Psychiatric Research Unit - WHO Collaborating Center for Mental Health, 2010) was used to assess well-being among the participants. The WHO-5 has five items to be answered on a 6-grade Likert scale ranging from “all the time” to “at no time”. Stress. Stress was measured with the Swedish version of the Perceived Stress Scale (PSS-10) (S. Cohen, Kamarck, & Mermelstein, 1983; Nordin & Nordin, 2013) consisting of ten items to be answered with a 5-grade Likert scale representing the range from “never” to “very often”. Anxiety. The Swedish version of the Generalized Anxiety Disorder 7-item scale (GAD-7) (Spitzer, Kroenke, Williams, & Lowe, 2006) was used to assess anxiety among the participants. The instrument consists of seven items to be answered with a 4-grade Likert scale ranging from “not at all” to “nearly every day”.Depression. Depression was measured using the Patient Health Questionnaire (PHQ9) (Kroenke, Spitzer, & Williams, 2001). The PHQ-9 is used in the Swedish healthcare as a screening instrument for depression and consist of nine items to be answered with a 4-grade Likert scale representing the range from “not at all” to “nearly every day”.Employment or return to education. The outcome was measured by questions on whether the participant was working, in education or in training (three different questions). The total time that the participants responded that they were either in employment, education or training was compiled. A total of 20 hours or more per week was considered “active”, less than 20 hours a week was regarded as “not active”."

Du redigerar ditt svar. Om du delar den här webbadressen kan även andra redigera ditt svar.

FYLL I ETT NYTT SVAR

6a-i) Online questionnaires: describe if they were validated for online use and apply CHERRIES items to describe how the questionnaires were designed/deployed

If outcomes were obtained through online questionnaires, describe if they were validated for online use and apply CHERRIES items to describe how the questionnaires were designed/deployed [9].

	1	2	3	4	5	
subitem not at all important	○	○	◉	○	○	essential

Rensa markering

Does your paper address subitem 6a-i?

Copy and paste relevant sections from manuscript text

Yes, the CHERRIE items are applied regarding design, IRB approval and informed consent process, recruitment process and survey administration. See for example the sections “Recruitment”, “Data collection and measures” and “Ethics approval and consent to participate” in the manuscript.

6a-ii) Describe whether and how “use” (including intensity of use/dosage) was defined/measured/monitored

Describe whether and how “use” (including intensity of use/dosage) was defined/measured/monitored (logins, logfile analysis, etc.). Use/adoption metrics are important process outcomes that should be reported in any ehealth trial.

	1	2	3	4	5	
subitem not at all important	○	○	○	◉	○	essential

Rensa markering

Does your paper address subitem 6a-ii?

Copy and paste relevant sections from manuscript text

Du redigerar ditt svar. Om du delar den här webbadressen kan även andra redigera ditt svar.

FYLL I ETT NYTT SVAR

6a-iii) Describe whether, how, and when qualitative feedback from participants was obtained

Describe whether, how, and when qualitative feedback from participants was obtained (e.g., through emails, feedback forms, interviews, focus groups).

	1	2	3	4	5	
subitem not at all important	○	○	○	◉	○	essential

Rensa markering

Does your paper address subitem 6a-iii?

Copy and paste relevant sections from manuscript text

Qualitative feedback from participants were not collected.

6b) Any changes to trial outcomes after the trial commenced, with reasons

Does your paper address CONSORT subitem 6b? *

Copy and paste relevant sections from the manuscript (include quotes in quotation marks "like this" to indicate direct quotes from your manuscript), or elaborate on this item by providing additional information not in the ms, or briefly explain why the item is not applicable/relevant for your study

No changes in trial outcomes were made after the trial commenced.

7a) How sample size was determined

NPT: When applicable, details of whether and how the clustering by care provides or centers was addressed

Du redigerar ditt svar. Om du delar den här webbadressen kan även andra redigera ditt svar.

FYLL I ETT NYTT SVAR

7a-i) Describe whether and how expected attrition was taken into account when calculating the sample size

Describe whether and how expected attrition was taken into account when calculating the sample size.

	1	2	3	4	5	
subitem not at all important	○	○	○	○	●	essential

Rensa markering

Does your paper address subitem 7a-i?

Copy and paste relevant sections from manuscript title (include quotes in quotation marks "like this" to indicate direct quotes from your manuscript), or elaborate on this item by providing additional information not in the ms, or briefly explain why the item is not applicable/relevant for your study

"Sample size was compiled based on a power of .80 and alpha .05 for a moderate effect in an analysis of variance with two groups resulting in a sample of 64 participants in each group (J. Cohen, 1992). The aim of recruitment was 180 participants to allow for a dropout of about 25%."

7b) When applicable, explanation of any interim analyses and stopping guidelines

Does your paper address CONSORT subitem 7b? *

Copy and paste relevant sections from the manuscript (include quotes in quotation marks "like this" to indicate direct quotes from your manuscript), or elaborate on this item by providing additional information not in the ms, or briefly explain why the item is not applicable/relevant for your study

No interim analyses were made during the trial.

8a) Method used to generate the random allocation sequence

[illegible]

Du redigerar ditt svar. Om du delar den här webbadressen kan även andra redigera ditt svar.

FYLL I ETT NYTT SVAR

Does your paper address CONSORT subitem 8a? *

Copy and paste relevant sections from the manuscript (include quotes in quotation marks "like this" to indicate direct quotes from your manuscript), or elaborate on this item by providing additional information not in the ms, or briefly explain why the item is not applicable/relevant for your study

"Randomization was performed 1:1 by the study coordinator using the statistical program SPSS by inserting the study id of the participants and using the command “Random sample of cases”, requesting that exactly 10 out of 20 to be selected. The randomization was performed three times, and the third randomization was used for all randomization rounds. "

8b) Type of randomisation; details of any restriction (such as blocking and block size)

Does your paper address CONSORT subitem 8b? *

Copy and paste relevant sections from the manuscript (include quotes in quotation marks "like this" to indicate direct quotes from your manuscript), or elaborate on this item by providing additional information not in the ms, or briefly explain why the item is not applicable/relevant for your study

"Randomization was performed continuously when 20 new participants had been recruited. This number was twice the number of participants that were required for a full potential group for the digital group meetings in the intervention group (described below). Randomization was performed 1:1 by the study coordinator using the statistical program SPSS by inserting the study id of the participants and using the command “Random sample of cases”, requesting that exactly 10 out of 20 to be selected. The randomization was performed three times, and the third randomization was used for all randomization rounds. Due to logistical reasons related to the timing of the group meetings, randomization was sometimes performed before reaching 20 new individuals. In those cases when an uneven number of individuals had been recruited, one additional person was requested to be assigned to the intervention group."

9) Mechanism used to implement the random allocation sequence (such as sequentially numbered containers), describing any steps taken to conceal the sequence until interventions were assigned

Du redigerar ditt svar. Om du delar den här webbadressen kan även andra redigera ditt svar.

FYLL I ETT NYTT SVAR

Does your paper address CONSORT subitem 9? *

Copy and paste relevant sections from the manuscript (include quotes in quotation marks "like this" to indicate direct quotes from your manuscript), or elaborate on this item by providing additional information not in the ms, or briefly explain why the item is not applicable/relevant for your study

"Randomization was performed 1:1 by the study coordinator using the statistical program SPSS by inserting the study id of the participants and using the command “Random sample of cases”, requesting that exactly 10 out of 20 to be selected. The randomization was performed three times, and the third randomization was used for all randomization rounds. "

10) Who generated the random allocation sequence, who enrolled participants, and who assigned participants to interventions

Does your paper address CONSORT subitem 10? *

Copy and paste relevant sections from the manuscript (include quotes in quotation marks "like this" to indicate direct quotes from your manuscript), or elaborate on this item by providing additional information not in the ms, or briefly explain why the item is not applicable/relevant for your study

The study coordinator generated the random allocation sequence and was the one to enroll the participants when having filed interest for the study. The study coordinator also assigned participants to the intervention or the control group in the online platform RedCap.

11a) If done, who was blinded after assignment to interventions (for example, participants, care providers, those assessing outcomes) and how

NPT: Whether or not administering co-interventions were blinded to group assignment

Du redigerar ditt svar. Om du delar den här webbadressen kan även andra redigera ditt svar.

FYLL I ETT NYTT SVAR

11a-i) Specify who was blinded, and who wasn't

Specify who was blinded, and who wasn't. Usually, in web-based trials it is not possible to blind the participants [1, 3] (this should be clearly acknowledged), but it may be possible to blind outcome assessors, those doing data analysis or those administering co-interventions (if any).

	1	2	3	4	5	
subitem not at all important	○	○	○	○	◉	essential

Rensa markering

Does your paper address subitem 11a-i? *

Copy and paste relevant sections from the manuscript (include quotes in quotation marks "like this" to indicate direct quotes from your manuscript), or elaborate on this item by providing additional information not in the ms, or briefly explain why the item is not applicable/relevant for your study

"Given the design of the study, neither the participants nor the group leaders could be blinded for the intervention conditions. Participants received information about the two conditions (intervention and control) and to which group they were assigned. Group leaders provided parts of the intervention and feedback, why they were aware of the condition. Researchers had access to the assessments made online."

11a-ii) Discuss e.g., whether participants knew which intervention was the "intervention of interest" and which one was the "comparator"

Informed consent procedures (4a-ii) can create biases and certain expectations - discuss e.g., whether participants knew which intervention was the "intervention of interest" and which one was the "comparator".

	1	2	3	4	5	
subitem not at all important	○	○	○	◉	○	essential

Rensa markering

Du redigerar ditt svar. Om du delar den här webbadressen kan även andra redigera ditt svar.

FYLL I ETT NYTT SVAR

Does your paper address subitem 11a-ii?

Copy and paste relevant sections from the manuscript (include quotes in quotation marks "like this" to indicate direct quotes from your manuscript), or elaborate on this item by providing additional information not in the ms, or briefly explain why the item is not applicable/relevant for your study

The participants were informed that they would either be assigned to test the app or to receive film clips.

11b) If relevant, description of the similarity of interventions

(this item is usually not relevant for ehealth trials as it refers to similarity of a placebo or sham intervention to a active medication/intervention)

Does your paper address CONSORT subitem 11b? *

Copy and paste relevant sections from the manuscript (include quotes in quotation marks "like this" to indicate direct quotes from your manuscript), or elaborate on this item by providing additional information not in the ms, or briefly explain why the item is not applicable/relevant for your study

We describe the intervention and control but it is clear to the participant which group s/he was assigned to, no placebo was used.

12a) Statistical methods used to compare groups for primary and secondary outcomes

NPT: When applicable, details of whether and how the clustering by care providers or centers was addressed

Du redigerar ditt svar. Om du delar den här webbadressen kan även andra redigera ditt svar.

FYLL I ETT NYTT SVAR

Does your paper address CONSORT subitem 12a? *

Copy and paste relevant sections from the manuscript (include quotes in quotation marks "like this" to indicate direct quotes from your manuscript), or elaborate on this item by providing additional information not in the ms, or briefly explain why the item is not applicable/relevant for your study

"A general linear model (GLM) was used to analyze the differences between t1 and t2 for the composite scores of well-being and psychological distress. Analyses were made using both per protocol (PP) and intention to treat (ITT) approach where missing values in the ITT analysis were replaced with series means of the intervention and control group. Differences in well-being and psychological distress (t2-t1) respectively were used as dependent variables in each model. Group, gender and disability were used as fixed factors in the GLM model. The interaction effect between those variables was included in the tables. Country of birth was not included as a fixed factor since the number of participants born abroad were few.Binary logistic regression was used for the analyses of activity status at t3."

12a-i) Imputation techniques to deal with attrition / missing values

Imputation techniques to deal with attrition / missing values: Not all participants will use the intervention/comparator as intended and attrition is typically high in ehealth trials. Specify how participants who did not use the application or dropped out from the trial were treated in the statistical analysis (a complete case analysis is strongly discouraged, and simple imputation techniques such as LOCF may also be problematic [4]).

	1	2	3	4	5	
subitem not at all important	○	○	○	○	◉	essential

Rensa markering

Does your paper address subitem 12a-i? *

Copy and paste relevant sections from the manuscript (include quotes in quotation marks "like this" to indicate direct quotes from your manuscript), or elaborate on this item by providing additional information not in the ms, or briefly explain why the item is not applicable/relevant for your study

"Analyses were made using both per protocol (PP) and intention to treat (ITT) approach where missing values in the ITT analysis were replaced with series means of the intervention and control group."

Du redigerar ditt svar. Om du delar den här webbadressen kan även andra redigera ditt svar.

FYLL I ETT NYTT SVAR

12b) Methods for additional analyses, such as subgroup analyses and adjusted analyses

Does your paper address CONSORT subitem 12b? *

Copy and paste relevant sections from the manuscript (include quotes in quotation marks "like this" to indicate direct quotes from your manuscript), or elaborate on this item by providing additional information not in the ms, or briefly explain why the item is not applicable/relevant for your study

"Differences in background data of the participants in the intervention and control groups were tested with Chi2 for categorical variables and Mann-Whitney U-test för continuous variables due to non-normality of the variables."

X26) REB/IRB Approval and Ethical Considerations [recommended as subheading under "Methods"] (not a CONSORT item)

X26-i) Comment on ethics committee approval

	1	2	3	4	5	
subitem not at all important	○	○	○	○	◉	essential

Rensa markering

Does your paper address subitem X26-i?

Copy and paste relevant sections from the manuscript (include quotes in quotation marks "like this" to indicate direct quotes from your manuscript), or elaborate on this item by providing additional information not in the ms, or briefly explain why the item is not applicable/relevant for your study

"The study was approved by the Swedish Ethical Review Authority (Dnr. 2020-03952). All participants were informed about the study and their rights and signed a digital informed consent form before participation. "

Du redigerar ditt svar. Om du delar den här webbadressen kan även andra redigera ditt svar.

FYLL I ETT NYTT SVAR

x26-ii) Outline informed consent procedures

Outline informed consent procedures e.g., if consent was obtained offline or online (how? Checkbox, etc.?), and what information was provided (see 4a-ii). See [6] for some items to be included in informed consent documents.

	1	2	3	4	5	
subitem not at all important	○	○	○	◉	○	essential

Rensa markering

Does your paper address subitem X26-ii?

Copy and paste relevant sections from the manuscript (include quotes in quotation marks "like this" to indicate direct quotes from your manuscript), or elaborate on this item by providing additional information not in the ms, or briefly explain why the item is not applicable/relevant for your study

"When a participant declared interest to participate in the study at this stage, s/he received an email with a link to a participant information and consent form, followed by a baseline questionnaire. " AND "Those who declared interest at this phase were sent a text message where they were asked if the study coordinator could phone them to give them more information about the study or if they preferred to be emailed."

X26-iii) Safety and security procedures

Safety and security procedures, incl. privacy considerations, and any steps taken to reduce the likelihood or detection of harm (e.g., education and training, availability of a hotline)

	1	2	3	4	5	
subitem not at all important	○	○	○	◉	○	essential

Rensa markering

Du redigerar ditt svar. Om du delar den här webbadressen kan även andra redigera ditt svar.

FYLL I ETT NYTT SVAR

Does your paper address subitem X26-iii?

Copy and paste relevant sections from the manuscript (include quotes in quotation marks "like this" to indicate direct quotes from your manuscript), or elaborate on this item by providing additional information not in the ms, or briefly explain why the item is not applicable/relevant for your study

"Participants who received high values on screening for depression, i.e., 15 points or more on the Patient Health Questionnaire (PHQ9) were excluded and provided contact details on where to seek care. The reason for exclusion was that depression was considered to need more treatment than this study intervention could provide. The excluded participants were also offered the chance to be contacted by any of the psychologists involved in the study if they wished to have further support. "

RESULTS

13a) For each group, the numbers of participants who were randomly assigned, received intended treatment, and were analysed for the primary outcome

NPT: The number of care providers or centers performing the intervention in each group and the number of patients treated by each care provider in each center

Does your paper address CONSORT subitem 13a? *

Copy and paste relevant sections from the manuscript (include quotes in quotation marks "like this" to indicate direct quotes from your manuscript), or elaborate on this item by providing additional information not in the ms, or briefly explain why the item is not applicable/relevant for your study

"A total of 590 young individuals declared interest in participating in the study (Figure 1). Of those, 193 individuals consented to participate in the study and filled in the baseline questionnaire. Due to high scores on the screening for depression, 42 individuals were excluded from the study and provided contact details to where to seek care. A total of 151 participants were randomized into either the intervention group (n=77) or the control group (n=74) and started the study." AND in the flowchart in Figure 1 it also describes how many in the intervention and the control group that answered the 2 follow-up questionnaires distributed post-intervention.

[illegible]

Du redigerar ditt svar. Om du delar den här webbadressen kan även andra redigera ditt svar.

FYLL I ETT NYTT SVAR

Does your paper address CONSORT subitem 13b? (NOTE: Preferably, this is shown in a CONSORT flow diagram) *

Copy and paste relevant sections from the manuscript (include quotes in quotation marks "like this" to indicate direct quotes from your manuscript), or elaborate on this item by providing additional information not in the ms, or briefly explain why the item is not applicable/relevant for your study

The information we have regarding losses and exclusions is the following: "Due to high scores on the screening for depression, 42 individuals were excluded from the study and provided contact details to where to seek care."

13b-i) Attrition diagram

Strongly recommended: An attrition diagram (e.g., proportion of participants still logging in or using the intervention/comparator in each group plotted over time, similar to a survival curve) or other figures or tables demonstrating usage/dose/engagement.

	1	2	3	4	5	
subitem not at all important	○	○	○	◉	○	essential

Rensa markering

Du redigerar ditt svar. Om du delar den här webbadressen kan även andra redigera ditt svar.

FYLL I ETT NYTT SVAR

Does your paper address subitem 13b-i?

Copy and paste relevant sections from the manuscript or cite the figure number if applicable (include quotes in quotation marks "like this" to indicate direct quotes from your manuscript), or elaborate on this item by providing additional information not in the ms, or briefly explain why the item is not applicable/relevant for your study

"Usage data from the app show that about seven out of ten participants in the intervention group downloaded and registered in the app (Table 3). Of those that registered, about 87% consented to having their usage followed. Slightly more than half of the participants did not complete any modules in the app. About 27% of the participants completed four or more modules and the same percentage participated in one or several digital group meetings. There were no significant differences in usage between those with disability and those without disability. Supplementary Table B displays some background and health related characteristics of the participants that completed any modules in the app and those that did not complete any. Among those that did not complete any modules, there was a higher proportion of participants being born outside of Sweden, a lower proportion of participants reporting that they trust other people in general and a higher proportion that were in contact with youth employment center or other activity towards NEET individuals. No significant differences were seen for the other characteristics."

14a) Dates defining the periods of recruitment and follow-up

Does your paper address CONSORT subitem 14a? *

Copy and paste relevant sections from the manuscript (include quotes in quotation marks "like this" to indicate direct quotes from your manuscript), or elaborate on this item by providing additional information not in the ms, or briefly explain why the item is not applicable/relevant for your study

"the first phase of the recruitment taking place in March and April 2021" AND "Phase two of the recruitment started in May 2021" AND "Data were collected using self-assessed electronically distributed questionnaires at three timepoints: baseline (t1), post-intervention (t2), and six months after randomization (t3)."

Du redigerar ditt svar. Om du delar den här webbadressen kan även andra redigera ditt svar.

FYLL I ETT NYTT SVAR

14a-i) Indicate if critical “secular events” fell into the study period

Indicate if critical “secular events” fell into the study period, e.g., significant changes in Internet resources available or “changes in computer hardware or Internet delivery resources”

	1	2	3	4	5	
subitem not at all important	○	○	◉	○	○	essential

Rensa markering

Does your paper address subitem 14a-i?

Copy and paste relevant sections from the manuscript (include quotes in quotation marks "like this" to indicate direct quotes from your manuscript), or elaborate on this item by providing additional information not in the ms, or briefly explain why the item is not applicable/relevant for your study

No critical secular events fell into the study period.

14b) Why the trial ended or was stopped (early)

Does your paper address CONSORT subitem 14b? *

Copy and paste relevant sections from the manuscript (include quotes in quotation marks "like this" to indicate direct quotes from your manuscript), or elaborate on this item by providing additional information not in the ms, or briefly explain why the item is not applicable/relevant for your study

The trial was not stopped early but ended when enough participants had been recruited.

15) A table showing baseline demographic and clinical characteristics for each group

NPT: When applicable, a description of care providers (case volume, qualification, expertise, etc.) and centers (volume) in each group

Du redigerar ditt svar. Om du delar den här webbadressen kan även andra redigera ditt svar.

FYLL I ETT NYTT SVAR

Does your paper address CONSORT subitem 15? *

Copy and paste relevant sections from the manuscript (include quotes in quotation marks "like this" to indicate direct quotes from your manuscript), or elaborate on this item by providing additional information not in the ms, or briefly explain why the item is not applicable/relevant for your study

Yes, Table 1 displays baseline demographic characteristics, self-reported health of the participants are reported in Supplementary Table A.

15-i) Report demographics associated with digital divide issues

In ehealth trials it is particularly important to report demographics associated with digital divide issues, such as age, education, gender, social-economic status, computer/Internet/ehealth literacy of the participants, if known.

	1	2	3	4	5	
subitem not at all important	○	○	◉	○	○	essential

Rensa markering

Du redigerar ditt svar. Om du delar den här webbadressen kan även andra redigera ditt svar.

FYLL I ETT NYTT SVAR

Does your paper address subitem 15-i? *

Copy and paste relevant sections from the manuscript (include quotes in quotation marks "like this" to indicate direct quotes from your manuscript), or elaborate on this item by providing additional information not in the ms, or briefly explain why the item is not applicable/relevant for your study

We do not report or discuss demographics associated with digital divide issues since the target group is relatively homogenous in terms of age. We do discuss the fact that the majority of participants were female and issues around that: "Focusing on the participants that were recruited, the larger proportion were females. This was a bit surprising since the gender division of NEETs in Sweden is relatively equal with a slightly higher representation of males (Eurofound, 2016b), and females have priorly been reported to be difficult to recruit (Myndigheten för ungdoms- och civilsamhällesfrågor, 2019). Male participants were also overrepresented among those that dropped out. A scoping review of the use of social media for recruitment to medical research studies did not find a gender difference in the recruited participants (Topolovec-Vranic & Natarajan, 2016) and Snapchat, which was the primary recruitment channel during the second recruitment phase when the majority of the participants were recruited has a relatively equal use over genders (Statista, 2023). However, a review of digital mental health interventions found that males were less likely to complete interventions compared to females and calls for interventions catering to the interests of young males (Garrido et al., 2019). It is possible that the intervention or the advertisement were more appealing to females even though the app and the short recruitment video were designed to appeal all genders. Gamified interventions have shown promise for promoting mental health across genders with an even greater potential effect on reduction in anxiety symptoms in samples with a higher proportions of male participants (Cheng & Ebrahimi, 2023). It is possible that using a gamified type of intervention could haveresulted in an increased recruitment and attainment of male participants."

16) For each group, number of participants (denominator) included in each analysis and whether the analysis was by original assigned groups

Du redigerar ditt svar. Om du delar den här webbadressen kan även andra redigera ditt svar.

FYLL I ETT NYTT SVAR

16-i) Report multiple “denominators” and provide definitions

Report multiple “denominators” and provide definitions: Report N’s (and effect sizes) “across a range of study participation [and use] thresholds” [1], e.g., N exposed, N consented, N used more than x times, N used more than y weeks, N participants “used” the intervention/comparator at specific pre-defined time points of interest (in absolute and relative numbers per group). Always clearly define “use” of the intervention.

	1	2	3	4	5	
subitem not at all important	○	○	○	○	◉	essential

Rensa markering

Does your paper address subitem 16-i? *

Copy and paste relevant sections from the manuscript (include quotes in quotation marks "like this" to indicate direct quotes from your manuscript), or elaborate on this item by providing additional information not in the ms, or briefly explain why the item is not applicable/relevant for your study

Yes, we provide denominators in all tables.

16-ii) Primary analysis should be intent-to-treat

Primary analysis should be intent-to-treat, secondary analyses could include comparing only “users”, with the appropriate caveats that this is no longer a randomized sample (see 18-i).

	1	2	3	4	5	
subitem not at all important	○	○	○	○	◉	essential

Rensa markering

Du redigerar ditt svar. Om du delar den här webbadressen kan även andra redigera ditt svar.

FYLL I ETT NYTT SVAR

Does your paper address subitem 16-ii?

Copy and paste relevant sections from the manuscript (include quotes in quotation marks "like this" to indicate direct quotes from your manuscript), or elaborate on this item by providing additional information not in the ms, or briefly explain why the item is not applicable/relevant for your study

We have made all analyses of the outcomes with both ITT and PP approach.

17a) For each primary and secondary outcome, results for each group, and the estimated effect size and its precision (such as 95% confidence interval)

Does your paper address CONSORT subitem 17a? *

Copy and paste relevant sections from the manuscript (include quotes in quotation marks "like this" to indicate direct quotes from your manuscript), or elaborate on this item by providing additional information not in the ms, or briefly explain why the item is not applicable/relevant for your study

Partial Eta Squared are presented for all GLM analyses.

17a-i) Presentation of process outcomes such as metrics of use and intensity of use

In addition to primary/secondary (clinical) outcomes, the presentation of process outcomes such as metrics of use and intensity of use (dose, exposure) and their operational definitions is critical. This does not only refer to metrics of attrition (13-b) (often a binary variable), but also to more continuous exposure metrics such as “average session length”. These must be accompanied by a technical description how a metric like a “session” is defined (e.g., timeout after idle time) [1] (report under item 6a).

	1	2	3	4	5	
subitem not at all important	○	○	○	◉	○	essential

Rensa markering

Du redigerar ditt svar. Om du delar den här webbadressen kan även andra redigera ditt svar.

FYLL I ETT NYTT SVAR

Does your paper address subitem 17a-i?

Copy and paste relevant sections from the manuscript (include quotes in quotation marks "like this" to indicate direct quotes from your manuscript), or elaborate on this item by providing additional information not in the ms, or briefly explain why the item is not applicable/relevant for your study

"Usage data from the app show that about seven out of ten participants in the intervention group downloaded and registered in the app (Table 3). Of those that registered, about 87% consented to having their usage followed. Slightly more than half of the participants did not complete any modules in the app. About 27% of the participants completed four or more modules and the same percentage participated in one or several digital group meetings. There were no significant differences in usage between those with disability and those without disability. "

17b) For binary outcomes, presentation of both absolute and relative effect sizes is recommended

Does your paper address CONSORT subitem 17b? *

Copy and paste relevant sections from the manuscript (include quotes in quotation marks "like this" to indicate direct quotes from your manuscript), or elaborate on this item by providing additional information not in the ms, or briefly explain why the item is not applicable/relevant for your study

This is not relevant for our data.

18) Results of any other analyses performed, including subgroup analyses and adjusted analyses, distinguishing pre-specified from exploratory

Does your paper address CONSORT subitem 18? *

Copy and paste relevant sections from the manuscript (include quotes in quotation marks "like this" to indicate direct quotes from your manuscript), or elaborate on this item by providing additional information not in the ms, or briefly explain why the item is not applicable/relevant for your study

Du redigerar ditt svar. Om du delar den här webbadressen kan även andra redigera ditt svar.

FYLL I ETT NYTT SVAR

18-i) Subgroup analysis of comparing only users

A subgroup analysis of comparing only users is not uncommon in ehealth trials, but if done, it must be stressed that this is a self-selected sample and no longer an unbiased sample from a randomized trial (see 16-iii).

	1	2	3	4	5	
subitem not at all important	○	○	○	○	◉	essential

Rensa markering

Does your paper address subitem 18-i?

Copy and paste relevant sections from the manuscript (include quotes in quotation marks "like this" to indicate direct quotes from your manuscript), or elaborate on this item by providing additional information not in the ms, or briefly explain why the item is not applicable/relevant for your study

Yes, additional analysis are made comparing the users with non-users in the intervention group.

19) All important harms or unintended effects in each group

(for specific guidance see CONSORT for harms)

Does your paper address CONSORT subitem 19? *

Copy and paste relevant sections from the manuscript (include quotes in quotation marks "like this" to indicate direct quotes from your manuscript), or elaborate on this item by providing additional information not in the ms, or briefly explain why the item is not applicable/relevant for your study

No important harms or unintended effects were detected during the trial.

Du redigerar ditt svar. Om du delar den här webbadressen kan även andra redigera ditt svar.

FYLL I ETT NYTT SVAR

19-i) Include privacy breaches, technical problems

Include privacy breaches, technical problems. This does not only include physical "harm" to participants, but also incidents such as perceived or real privacy breaches [1], technical problems, and other unexpected/unintended incidents. "Unintended effects" also includes unintended positive effects [2].

	1	2	3	4	5	
subitem not at all important	○	○	○	●	○	essential

Rensa markering

Does your paper address subitem 19-i?

Copy and paste relevant sections from the manuscript (include quotes in quotation marks "like this" to indicate direct quotes from your manuscript), or elaborate on this item by providing additional information not in the ms, or briefly explain why the item is not applicable/relevant for your study

No privacy breaches, technical problems or other unexpected incidents were detected during the trial.

19-ii) Include qualitative feedback from participants or observations from staff/researchers

Include qualitative feedback from participants or observations from staff/researchers, if available, on strengths and shortcomings of the application, especially if they point to unintended/unexpected effects or uses. This includes (if available) reasons for why people did or did not use the application as intended by the developers.

	1	2	3	4	5	
subitem not at all important	○	○	●	○	○	essential

Rensa markering

Du redigerar ditt svar. Om du delar den här webbadressen kan även andra redigera ditt svar.

FYLL I ETT NYTT SVAR

Does your paper address subitem 19-ii?

Copy and paste relevant sections from the manuscript (include quotes in quotation marks "like this" to indicate direct quotes from your manuscript), or elaborate on this item by providing additional information not in the ms, or briefly explain why the item is not applicable/relevant for your study

We did not include qualitative feedback from participants and have included this as a limitation in the manuscript: "Furthermore, the fact that only quantitative data were collected limits the possibilities of providing explanations of the use and non use of the app or for potential reasons for dropping out."

DISCUSSION

22) Interpretation consistent with results, balancing benefits and harms, and considering other relevant evidence

NPT: In addition, take into account the choice of the comparator, lack of or partial blinding, and unequal expertise of care providers or centers in each group

22-i) Restate study questions and summarize the answers suggested by the data, starting with primary outcomes and process outcomes (use)

Restate study questions and summarize the answers suggested by the data, starting with primary outcomes and process outcomes (use).

	1	2	3	4	5	
subitem not at all important	○	○	○	◉	○	essential

Rensa markering

Du redigerar ditt svar. Om du delar den här webbadressen kan även andra redigera ditt svar.

FYLL I ETT NYTT SVAR

Does your paper address subitem 22-i? *

Copy and paste relevant sections from the manuscript (include quotes in quotation marks "like this" to indicate direct quotes from your manuscript), or elaborate on this item by providing additional information not in the ms, or briefly explain why the item is not applicable/relevant for your study

"The study aimed to expand the knowledge on the effects in terms of well-being, psychological distress and activity level of an app-based intervention on NEETs with and without disability. The main findings of the study are that no differences were seen overall between the intervention and control groups, irrespective of whether the participants had completed a module in the app or not. No significant differences were found regarding the effect on being active 20 hours or more per week six months post randomization except for within the intervention group where those that had completed one or several modules in the intervention were more likely to be active compared to those that did not complete any. "

22-ii) Highlight unanswered new questions, suggest future research

Highlight unanswered new questions, suggest future research.

	1	2	3	4	5	
subitem not at all important	○	○	○	◉	○	essential

Rensa markering

Does your paper address subitem 22-ii?

Copy and paste relevant sections from the manuscript (include quotes in quotation marks "like this" to indicate direct quotes from your manuscript), or elaborate on this item by providing additional information not in the ms, or briefly explain why the item is not applicable/relevant for your study

"There might be a need to include other social media sources for recruitment and to adjust the app and its content to increase the chances of including and retaining male participants since they were both underrepresented in the study population and overrepresented among those that dropped out. NEETs with disability are of particular concern and might need additional efforts or other types of interventions than the one investigated herein."

Du redigerar ditt svar. Om du delar den här webbadressen kan även andra redigera ditt svar.

FYLL I ETT NYTT SVAR

20) Trial limitations, addressing sources of potential bias, imprecision, and, if relevant, multiplicity of analyses

20-i) Typical limitations in ehealth trials

Typical limitations in ehealth trials: Participants in ehealth trials are rarely blinded. Ehealth trials often look at a multiplicity of outcomes, increasing risk for a Type I error. Discuss biases due to non-use of the intervention/usability issues, biases through informed consent procedures, unexpected events.

	1	2	3	4	5	
subitem not at all important	○	○	○	○	◉	essential

Rensa markering

Does your paper address subitem 20-i? *

Copy and paste relevant sections from the manuscript (include quotes in quotation marks "like this" to indicate direct quotes from your manuscript), or elaborate on this item by providing additional information not in the ms, or briefly explain why the item is not applicable/relevant for your study

"Among the strengths of the study is the use of an RCT design using an active control group which lay ground for an investigation of potential additional benefits of the specific app. The downside of having an active control group is that it make it more difficult to detect effects (Bennett et al., 2020; Klimczak et al., 2023; Linardon, 2020). Furthermore, the analyses made with both PP and ITT approach to handle missing data in the material resulted in similar results in all analyses. Missing data due to a drop-out exceeding the expected 20% and with limited possibilities to find out the reasons why are major limitations in the study that lower the power and makes it difficult to draw firm conclusions. Apart from a high drop-out, there were also problems with low adherence to the intervention and low participation in the digital group meetings. About one in ten participants were born outside of Sweden which is about half the share compared to the Swedish setting where one out of five in the age group are born outside of Sweden (Statistics Sweden, 2024) and the numbers are to small in this sample to allow for assessing the effect of ethnicity which could have provided additional insights of the app's effects."

Du redigerar ditt svar. Om du delar den här webbadressen kan även andra redigera ditt svar.

FYLL I ETT NYTT SVAR

21) Generalisability (external validity, applicability) of the trial findings

NPT: External validity of the trial findings according to the intervention, comparators, patients, and care providers or centers involved in the trial

21-i) Generalizability to other populations

Generalizability to other populations: In particular, discuss generalizability to a general Internet population, outside of a RCT setting, and general patient population, including applicability of the study results for other organizations

	1	2	3	4	5	
subitem not at all important	○	○	○	○	◉	essential

Rensa markering

Does your paper address subitem 21-i?

Copy and paste relevant sections from the manuscript (include quotes in quotation marks "like this" to indicate direct quotes from your manuscript), or elaborate on this item by providing additional information not in the ms, or briefly explain why the item is not applicable/relevant for your study

We discuss the population recruited in relation to the general population, for example the large proportion of females and the high proportion of participants with a disability.

21-ii) Discuss if there were elements in the RCT that would be different in a routine application setting

Discuss if there were elements in the RCT that would be different in a routine application setting (e.g., prompts/reminders, more human involvement, training sessions or other co-interventions) and what impact the omission of these elements could have on use, adoption, or outcomes if the intervention is applied outside of a RCT setting.

	1	2	3	4	5	
subitem not at all important	○	○	○	○	◉	essential

Rensa markering

Du redigerar ditt svar. Om du delar den här webbadressen kan även andra redigera ditt svar. FYLL I ETT NYTT SVAR

Does your paper address subitem 21-ii?

Copy and paste relevant sections from the manuscript (include quotes in quotation marks "like this" to indicate direct quotes from your manuscript), or elaborate on this item by providing additional information not in the ms, or briefly explain why the item is not applicable/relevant for your study

If this app would have showed to be effective, the routine application setting roll out would appear similar as in the trial.

OTHER INFORMATION

23) Registration number and name of trial registry

Does your paper address CONSORT subitem 23? *

Copy and paste relevant sections from the manuscript (include quotes in quotation marks "like this" to indicate direct quotes from your manuscript), or elaborate on this item by providing additional information not in the ms, or briefly explain why the item is not applicable/relevant for your study

"Trial registration: Registered on 12 February 2021 at ISRCTN (#ISRCTN46697028), https://doi.org/10.1186/ISRCTN46697028 "

24) Where the full trial protocol can be accessed, if available

Does your paper address CONSORT subitem 24? *

Cite a Multimedia Appendix, other reference, or copy and paste relevant sections from the manuscript (include quotes in quotation marks "like this" to indicate direct quotes from your manuscript), or elaborate on this item by providing additional information not in the ms, or briefly explain why the item is not applicable/relevant for your study

The full trial protocol is not available but trial information is found in the trial registry.

Du redigerar ditt svar. Om du delar den här webbadressen kan även andra redigera ditt svar.

FYLL I ETT NYTT SVAR

25) Sources of funding and other support (such as supply of drugs), role of funders

Does your paper address CONSORT subitem 25? *

Copy and paste relevant sections from the manuscript (include quotes in quotation marks "like this" to indicate direct quotes from your manuscript), or elaborate on this item by providing additional information not in the ms, or briefly explain why the item is not applicable/relevant for your study

Source of funding is found in the Acknowledgement section: "Last but not least, we would like to thank the Swedish Research Council for Health, Working Life and Welfare (Forte) who funded the study (2015-01136). The funders had no role in the design of the study or the reporting of the results."

X27) Conflicts of Interest (not a CONSORT item)

X27-i) State the relation of the study team towards the system being evaluated

In addition to the usual declaration of interests (financial or otherwise), also state the relation of the study team towards the system being evaluated, i.e., state if the authors/evaluators are distinct from or identical with the developers/sponsors of the intervention.

	1	2	3	4	5	
subitem not at all important	○	○	○	○	◉	essential

Rensa markering

Du redigerar ditt svar. Om du delar den här webbadressen kan även andra redigera ditt svar.

FYLL I ETT NYTT SVAR

Does your paper address subitem X27-i?

Copy and paste relevant sections from the manuscript (include quotes in quotation marks "like this" to indicate direct quotes from your manuscript), or elaborate on this item by providing additional information not in the ms, or briefly explain why the item is not applicable/relevant for your study

"FL and JR have positions both at Karolinska Institutet and at the non-profitable foundation 29k that developed the app. Both FL and JR have participated in the development and the content of the platform 29k as employed at 29k. Neither FL or JR were involved in the analysis of the data and the foundation 29k have not been involved either in the design of the study or in the manuscript writing. LB and LL were in charge of the analysis and have no competing interests."

About the CONSORT EHEALTH checklist

As a result of using this checklist, did you make changes in your manuscript? *

- ○ yes, major changes
- ◉ yes, minor changes
- ○ no

What were the most important changes you made as a result of using this checklist?

Adding information in the methods section.

How much time did you spend on going through the checklist INCLUDING making changes in your manuscript *

Approximately 8-10 hours.

Du redigerar ditt svar. Om du delar den här webbadressen kan även andra redigera ditt svar.

FYLL I ETT NYTT SVAR

As a result of using this checklist, do you think your manuscript has improved? *

- (x) yes
- ( ) no
- ( ) Övrigt:

Would you like to become involved in the CONSORT EHEALTH group?

This would involve for example becoming involved in participating in a workshop and writing an "Explanation and Elaboration" document

- ( ) yes
- (x) no
- ( ) Övrigt:

Rensa markering

Any other comments or questions on CONSORT EHEALTH

The checklist is extensive and time consuming which could maybe be improved with some changes in the layout that make it clearer what is the question and the comments around the question about how to answer each question.

STOP - Save this form as PDF before you click submit

To generate a record that you filled in this form, we recommend to generate a PDF of this page (on a Mac, simply select "print" and then select "print as PDF") before you submit it.

When you submit your (revised) paper to JMIR, please upload the PDF as supplementary file.

Don't worry if some text in the textboxes is cut off, as we still have the complete information in our database. Thank you!

Du redigerar ditt svar. Om du delar den här webbadressen kan även andra redigera ditt svar.

FYLL I ETT NYTT SVAR

Final step: Click submit !

Click submit so we have your answers in our database!

Skicka

Skicka aldrig lösenord med Google Formulär

Det här innehållet har varken skapats eller godkänts av Google. - Användarvillkor - Integritetspolicy

Does this form look suspicious? Rapport

Google Formulär

Du redigerar ditt svar. Om du delar den här webbadressen kan även andra redigera ditt svar.

FYLL I ETT NYTT SVAR

Du redigerar ditt svar. Om du delar den här webbadressen kan även andra redigera ditt svar.

FYLL I ETT NYTT SVAR
