# Supplementary material for: Effects of an App-Based Intervention on Psychological Well-Being Among Young Individuals not in Employment, Education, or Training With and Those Without Disability: Subgroup Analysis of a Randomized Controlled Trial
Source: JMIR Pediatr Parent. 2026 Feb 12;9:e71367. doi: 10.2196/71367 (PMC12946780; doi:10.2196/71367)
Supplement: Multimedia Appendix 2 [file pediatrics_v9i1e71367_app2.docx]

Links to the film clips sent to the control group

Stress part 1, what is stress and how does it feels, 6 minutes:

[https://www.youtube.com/watch?v=dCYPHFr5pqk](https://eur01.safelinks.protection.outlook.com/?url=https%3A%2F%2Fwww.youtube.com%2Fwatch%3Fv%3DdCYPHFr5pqk&data=04%7C01%7Clene.lindberg%40ki.se%7Ce820f1abf90b408c70d908d8da806fc7%7Cbff7eef1cf4b4f32be3da1dda043c05d%7C0%7C0%7C637499592459167626%7CUnknown%7CTWFpbGZsb3d8eyJWIjoiMC4wLjAwMDAiLCJQIjoiV2luMzIiLCJBTiI6Ik1haWwiLCJXVCI6Mn0%3D%7C1000&sdata=WGkwRkOqjkg7%2FCZOlD1hdrXRziYlCVSxrEEEAIDs4es%3D&reserved=0)

Stress part 2, how to handle stress, 5 minutes:

[https://www.youtube.com/watch?v=4kpr3OwEZwI](https://eur01.safelinks.protection.outlook.com/?url=https%3A%2F%2Fwww.youtube.com%2Fwatch%3Fv%3D4kpr3OwEZwI&data=04%7C01%7Clene.lindberg%40ki.se%7Ce820f1abf90b408c70d908d8da806fc7%7Cbff7eef1cf4b4f32be3da1dda043c05d%7C0%7C0%7C637499592459167626%7CUnknown%7CTWFpbGZsb3d8eyJWIjoiMC4wLjAwMDAiLCJQIjoiV2luMzIiLCJBTiI6Ik1haWwiLCJXVCI6Mn0%3D%7C1000&sdata=%2BUTh89oBBVvfeKGZuGinLR%2FoUem8a4UkhMteam4Jxns%3D&reserved=0)

Mental ill-health part 1, what is mental ill-health, 8 minutes:

[https://www.youtube.com/watch?v=9Q0ssASQpUk&t=48s](https://eur01.safelinks.protection.outlook.com/?url=https%3A%2F%2Fwww.youtube.com%2Fwatch%3Fv%3D9Q0ssASQpUk%26t%3D48s&data=04%7C01%7Clene.lindberg%40ki.se%7Ce820f1abf90b408c70d908d8da806fc7%7Cbff7eef1cf4b4f32be3da1dda043c05d%7C0%7C0%7C637499592459177584%7CUnknown%7CTWFpbGZsb3d8eyJWIjoiMC4wLjAwMDAiLCJQIjoiV2luMzIiLCJBTiI6Ik1haWwiLCJXVCI6Mn0%3D%7C1000&sdata=DXVTffK9SGHEVPWIOphlQBFjnN1GT%2F0ev05MlTxV%2Ft4%3D&reserved=0)

Mental ill-health part 2, how to handle mental ill-health, 4 minutes:

[https://www.youtube.com/watch?v=6l-CVbv3gGc](https://eur01.safelinks.protection.outlook.com/?url=https%3A%2F%2Fwww.youtube.com%2Fwatch%3Fv%3D6l-CVbv3gGc&data=04%7C01%7Clene.lindberg%40ki.se%7Ce820f1abf90b408c70d908d8da806fc7%7Cbff7eef1cf4b4f32be3da1dda043c05d%7C0%7C0%7C637499592459177584%7CUnknown%7CTWFpbGZsb3d8eyJWIjoiMC4wLjAwMDAiLCJQIjoiV2luMzIiLCJBTiI6Ik1haWwiLCJXVCI6Mn0%3D%7C1000&sdata=YZniy9Zu6ZnRFVbbVi4i1oaW1PwGwB9ZZz9b416LfBQ%3D&reserved=0)

Mental ill-health part 3, how is the healthcare, 5 minutes:

[https://www.youtube.com/watch?v=JiaDy8__fGU](https://eur01.safelinks.protection.outlook.com/?url=https%3A%2F%2Fwww.youtube.com%2Fwatch%3Fv%3DJiaDy8__fGU&data=04%7C01%7Clene.lindberg%40ki.se%7Ce820f1abf90b408c70d908d8da806fc7%7Cbff7eef1cf4b4f32be3da1dda043c05d%7C0%7C0%7C637499592459177584%7CUnknown%7CTWFpbGZsb3d8eyJWIjoiMC4wLjAwMDAiLCJQIjoiV2luMzIiLCJBTiI6Ik1haWwiLCJXVCI6Mn0%3D%7C1000&sdata=hotpAKHzurAY6sdn0T%2BU01i%2B0LbQEuioUIXhmLeNlJ4%3D&reserved=0)

Self-esteem and the purpose of life, 7 minutes:

[https://www.youtube.com/watch?v=5JBOFw9i_GM](https://eur01.safelinks.protection.outlook.com/?url=https%3A%2F%2Fwww.youtube.com%2Fwatch%3Fv%3D5JBOFw9i_GM&data=04%7C01%7Clene.lindberg%40ki.se%7Ce820f1abf90b408c70d908d8da806fc7%7Cbff7eef1cf4b4f32be3da1dda043c05d%7C0%7C0%7C637499592459187540%7CUnknown%7CTWFpbGZsb3d8eyJWIjoiMC4wLjAwMDAiLCJQIjoiV2luMzIiLCJBTiI6Ik1haWwiLCJXVCI6Mn0%3D%7C1000&sdata=E8JLEc8wvc4h8JMfKYPHMgnXTBv4rN%2Borj2uoVW9KFw%3D&reserved=0)
