# Supplementary material for: Effects of an App-Based Intervention on Psychological Well-Being Among Young Individuals not in Employment, Education, or Training With and Those Without Disability: Subgroup Analysis of a Randomized Controlled Trial
Source: JMIR Pediatr Parent. 2026 Feb 12;9:e71367. doi: 10.2196/71367 (PMC12946780; doi:10.2196/71367)
Supplement: Multimedia Appendix 3 [file pediatrics_v9i1e71367_app3.docx]

**Supplementary Table A.** Self-reported health of the participants at baseline

|  | |  | **ARM** | | | **REPORTED DISABILITY** | | |
| --- | --- | --- | --- | --- | --- | --- | --- | --- |
|  | | **All**  **(N=151)** | **Intervention**  **(N=77)** | **Control**  **(N=74)** | ***χ2 or M-W*** | **Disability**  **(N=75)** | **No disability**  **(N=76)** | ***χ2 or M-W*** |
|  | | n (%) | n (%) | n (%) | *P* | n (%) | n (%) | *P* |
| **Well-being (WHO-5)**, mean (sd) | | 45.7 (16.9) | 45.9 (16.3) | 45.5 (17.5) | *.88* | 43.3 (15.0) | 48.2 (18.3) | *.14* |
| **Self-esteem (Rosenberg),** mean (sd) | | 14.5 (6.3) | 15.1 (5.7) | 13.9 (6.8) | *.11* | 12.0 (5.6) | 16.1 (6.5) | *.01* |
| **Anxiety (GAD-7)** | |  |  |  |  |  |  |  |
|  | Minimal anxiety (0-4) | 34 (22.5) | 20 (26.0) | 14 (18.9) | *.28* | 12 (16.0) | 22 (28.9) | *.13* |
|  | Mild anxiety (5-9) | 74 (49.7) | 40 (51.9) | 34 (45.9) |  | 37 (49.3) | 37 (48.7) |  |
|  | Moderate or severe anxiety (10-21) | 41 (27.2) | 17 (22.1) | 24 (32.4) |  | 24 (32.0) | 17 (22.4) |  |
|  | Information missing | * (*) | - | *(*) |  | *(*) | - |  |
| **Relation to mother** | |  |  |  |  |  |  |  |
|  | Very good or good | 105 (69.5) | 54 (70.1) | 51 (68.9) | *.82* | 52 (69.3) | 53 (69.7) | *.96* |
|  | Ok to very bad or no answer | 46 (30.5) | 23 (29.9) | 23 (31.1) |  | 23 (30.7) | 23 (30.3) |  |
| **Relation to father** | |  |  |  |  |  |  |  |
|  | Very good or good | 62 (41.1) | 32 (41.6) | 30 (40.5) | *.90* | 28 (37.3) | 34 (44.7) | *.36* |
|  | Ok to very bad or no answer | 89 (58.9) | 45 (58.4) | 44 (59.5) |  | 47 (62.7) | 42 (55.3) |  |
| **Trust other people in general** | |  |  |  |  |  |  |  |
|  | Yes | 80 (53.0) | 42 (54.5) | 38 (51.4) | *.69* | 40 (53.3) | 40 (52.6) | *.93* |
|  | No | 71 (47.0) | 35 (45.5) | 36 (48.6) |  | 35 (46.7) | 36 (47.4) |  |
| **Trust in institutions*** | |  |  |  |  |  |  |  |
|  | None or one | 52 (34.4) | 24 (31.2) | 28 (37.8) | *.39* | 29 (38.7) | 23 (30.3) | *.28* |
|  | Two or more | 99 (65.6) | 53 (68.8) | 46 (62.2) |  | 46 (61.3) | 53 (69.7) |  |
| **Stress (PSS-10)** | |  |  |  |  |  |  |  |
|  | Low level (0-13) | 18 (11.9) | 11 (14.3) | 7 (9.5) | *.69* | 5 (6.7) | 13 (17.1) | *.06* |
|  | Moderate (14-26) | 113 (74.8) | 57 (74.0) | 56 (75.7) |  | 58 (77.3) | 55 (72.4) |  |
|  | High (27-40) | 18 (11.9) | 9 (11.7) | 9 (12.2) |  | 12 (16.0) | 6 (7.9) |  |
|  | Information missing | * (*) | - | * (*) |  | - | *(*) |  |
| **Depression (PHQ-9)** | |  |  |  |  |  |  |  |
|  | No/minimal depr. symptoms (≤4) | 18 (11.9) | 10 (13.0) | 8 (10.8) | *.82* | 6 (8.0) | 12 (15.8) | *.21* |
|  | Mild depr. symptoms (5-9) | 47 (31.1) | 25 (32.5) | 22 (29.7) |  | 27 (36.0) | 20 (26.3) |  |
|  | Moderate depr. symptoms (10-14) | 86 (57.0) | 42 (54.5) | 44 (59.5) |  | 42 (56.0) | 44 (57.9) |  |

*Police, Healthcare, School, Employment office, Social services
