# Supplementary material for: Effects of an App-Based Intervention on Psychological Well-Being Among Young Individuals not in Employment, Education, or Training With and Those Without Disability: Subgroup Analysis of a Randomized Controlled Trial
Source: JMIR Pediatr Parent. 2026 Feb 12;9:e71367. doi: 10.2196/71367 (PMC12946780; doi:10.2196/71367)
Supplement: Multimedia Appendix 4 [file pediatrics_v9i1e71367_app4.docx]

**Supplementary Table B**. Background and health related characteristics of participants in the intervention group according to whether they completed modules in the app or not

|  | | **INTERVENTION** | | |
| --- | --- | --- | --- | --- |
|  | | **Completed one or more modules**  **(N=34)** | **Did not complete any modules**  **(N=43)** | ***χ2 or M-W*** |
|  | | n (%) | n (%) | *P* |
| **Gender** | |  |  |  |
|  | Women | 27 (79.4) | 29 (69.0) | *.31* |
|  | Men | 7 (20.6) | 13 (31.0) |  |
| **Age** | |  |  |  |
|  | 16-19 | 11 (32.4) | 19 (44.2) | *.29* |
|  | 20-24 | 23 (67.6) | 24 (55.8) |  |
| **Birth country** | |  |  |  |
|  | Sweden | 34 (100.0) | 36 (83.7) | *.01* |
|  | Other than Sweden | 0 (0.0) | 7 (16.3) |  |
| **Trust institutions** | |  |  |  |
|  | None or one | 7 (20.6) | 17 (39.5) | *.08* |
|  | Two or several | 27 (79.4) | 26 (60.5) |  |
| **Trust people in general** | |  |  |  |
|  | Yes | 23 (67.6) | 19 (44.2) | *.04* |
|  | No | 11 (32.4) | 24 (55.8) |  |
| **Disability** | |  |  |  |
|  | Yes | 18 (52.9) | 23 (53.5) | *.96* |
|  | No | 16 (47.1) | 20 (46.5) |  |
| **Relation to mother** | |  |  |  |
|  | Very good or good | 25 (73.5) | 29 (67.4) | *.56* |
|  | Ok to very bad | 9 (26.5) | 14 (32.6) |  |
| **Relation to father** | |  |  |  |
|  | Very good or good | 13 (38.2) | 19 (44.2) | *.60* |
|  | Ok to very bad | 21 (61.8) | 24 (55.8) |  |
| **Depression (PHQ-9)** | |  |  |  |
|  | No/minimal depr. symptoms (≤4) | 4 (11.8) | 6 (14.0) | *.88* |
|  | Mild depr. symptoms (5-9) | 12 (35.3) | 13 (30.2) |  |
|  | Moderate depr. symptoms (10-14) | 18 (52.9) | 24 (55.8) |  |
| **Anxiety (GAD-7)** | |  |  |  |
|  | Minimal anxiety (0-4) | 9 (26.5) | 11 (25.6) | *.70* |
|  | Mild anxiety (5-9) | 19 (55.9) | 21 (48.8) |  |
|  | Moderate or severe anxiety (10-21) | 6 (17.6) | 11 (25.6) |  |
| **Stress (PSS-10)** | |  |  |  |
|  | Low level (0-13) | 5 (14.7) | 6 (14.0) | *.79* |
|  | Moderate (14-26) | 26 (76.5) | 31 (72.1) |  |
|  | High (27-40) | 3 (8.8) | 6 (14.0) |  |
| **WHO**, mean (sd) | | 44.5 (16.5) | 47.1 (16.3) | *.40* |
| **Rosenberg**, mean (sd) | | 14.5 (5.8) | 15.7 (5.7) | *.22* |
| **Graduated 9^th^ grade** | |  |  |  |
|  | Yes | 34 (100.0) | 40 (93.0) | *.25* |
|  | No or don’t know | 0 (0.0) | 3 (7.0) |  |
| **Completed high school** | |  |  |  |
|  | Yes | 22 (64.7) | 22 (51.2) | *.23* |
|  | No | 12 (35.3) | 21 (48.8) |  |
| **Time in NEET status** | |  |  |  |
|  | 0-2 months | 10 (31.3) | 7 (16.7) | *.33* |
|  | 3-12 months | 16 (50.0) | 25 (59.5) |  |
|  | 13 months or more | 6 (18.8) | 10 (23.8) |  |
|  | Information missing | * (*) | * (*) |  |
| **Contact with youth employment center or other activity working with NEETs** | |  |  |  |
|  | Yes | 3 (8.8) | 15 (34.9) | *.01* |
|  | No | 31 (91.2) | 28 (65.1) |  |
| **In a work training program or similar** | |  |  |  |
|  | Yes | 4 (11.8) | 9 (20.9) | *.29* |
|  | No | 30 (88.2) | 34 (79.1) |  |
| **Prior work experience** | |  |  |  |
|  | Yes | 26 (76.5) | 27 (62.8) | *.20* |
|  | No | 8 (23.5) | 16 (37.2) |  |
